# COCO-Search18 fixation dataset for predicting goal-directed attention control

**DOI:** 10.1038/s41598-021-87715-9

**Published:** 2021-04-22

**Authors:** Yupei Chen, Zhibo Yang, Seoyoung Ahn, Dimitris Samaras, Minh Hoai, Gregory Zelinsky

**Affiliations:** 1 Department of Psychology, Stony Brook University, New York, USA; 2 Department of Computer Science, Stony Brook University, New York, USA

**Keywords:** Psychology, Human behaviour

## Abstract

Attention control is a basic behavioral process that has been studied for decades. The currently best models of attention control are deep networks trained on free-viewing behavior to predict bottom-up attention control – saliency. We introduce COCO-Search18, the first dataset of laboratory-quality *goal-directed behavior* large enough to train deep-network models. We collected eye-movement behavior from 10 people searching for each of 18 target-object categories in 6202 natural-scene images, yielding $\sim$ 300,000 search fixations. We thoroughly characterize COCO-Search18, and benchmark it using three machine-learning methods: a ResNet50 object detector, a ResNet50 trained on fixation-density maps, and an inverse-reinforcement-learning model trained on behavioral search scanpaths. Models were also trained/tested on images transformed to approximate a foveated retina, a fundamental biological constraint. These models, each having a different reliance on behavioral training, collectively comprise the new state-of-the-art in predicting goal-directed search fixations. Our expectation is that future work using COCO-Search18 will far surpass these initial efforts, finding applications in domains ranging from human-computer interactive systems that can anticipate a person’s intent and render assistance to the potentially early identification of attention-related clinical disorders (ADHD, PTSD, phobia) based on deviation from neurotypical fixation behavior.

The control of visual attention comes broadly in two forms. One is bottom-up, where control is exerted purely by the visual input^1,2^. This is the form of attention predicted by saliency models, which exploded in popularity in the behavioral fixation-prediction and computer-vision literatures^2–5^. The other form of control is top-down, where behavioral goals rather than bottom-up salience control the allocation of visual attention. Goal-directed attention control underlies all the things that we *try* to do, and this diversity makes its prediction vastly more challenging than predicting bottom-up saliency, and more important. In addition to its basic research value, a better understanding of goal-directed attention could lead to the development of biomarkers for neurotypical attention behavior against which clinical conditions can be quantitatively compared, and to advances in intelligent human-computer interactive systems that can anticipate a user’s visual goals and render real-time assistance^6–8^.

Goal-directed attention has been studied for decades^9–16^, largely in the context of visual search. Search is arguably the most basic of goal-directed behaviors; there is a target object and the goal is to find it, or conclude its absence. Goals are extremely effective in controlling the allocation of gaze. Imagine two encounters with a kitchen, first with the goal of learning the time from a wall clock and again with the goal of warming a cup of coffee. These “clock” and “microwave” searches would yield two very different patterns of eye movement, as recently demonstrated in a test of this gedanken experiment^17^, and understanding this goal-directed control has been a core aim of search theory. The visual search literature is itself voluminous (see reviews^18–20^). Here we focus on the prediction of image locations that people fixate as they search for objects, and how the selection of these fixation locations depends on the target goal.

The visual search literature is not only mature in its empirical work, it is also rich with many hugely influential theories and models^10–13,15,21^. Yet despite this success, over the last years progress has stalled. Our premise is that this is due to the absence of a dataset of search behavior sufficiently large to train deep network models. Our belief is based on observation of what occurred in the bottom-up attention-control literature during the same time. The prediction of fixations during free viewing, the task-less cousin of visual search, has become an extremely active research topic, complete with managed competitions and leaderboards for the most predictive models^22^ (http://saliency.mit.edu/). The best of these saliency models are all deep networks, and to our point, all of them were trained on large datasets of labeled human behavior^23–27^. For example, one of the best of these models, DeepGaze II^26^, is a deep network pre-trained on SALICON^25^. SALICON is a crowd-sourced dataset consisting of images that were annotated with mouse-based data approximating the attention shifts made during free viewing. This model of fixation prediction during free viewing was therefore trained on a form of free-viewing behavior. Without SALICON, DeepGaze II, and models like it^23–25,27^, would not have been possible, and our understanding of free-viewing behavior, widely believed to reflect bottom-up attention control, would be greatly diminished. For the task of visual search, there is nothing remotely comparable to SALICON^25^. Here we describe in detail COCO-Search18, the largest dataset of goal-directed search fixations in the world. COCO-Search18 was recently introduced at CVPR2020^28^, and our aim in this paper is to elaborate on the richness of this dataset so as to increase its usefulness to researchers interesting in modeling top-down attention control.

**Figure 1:**
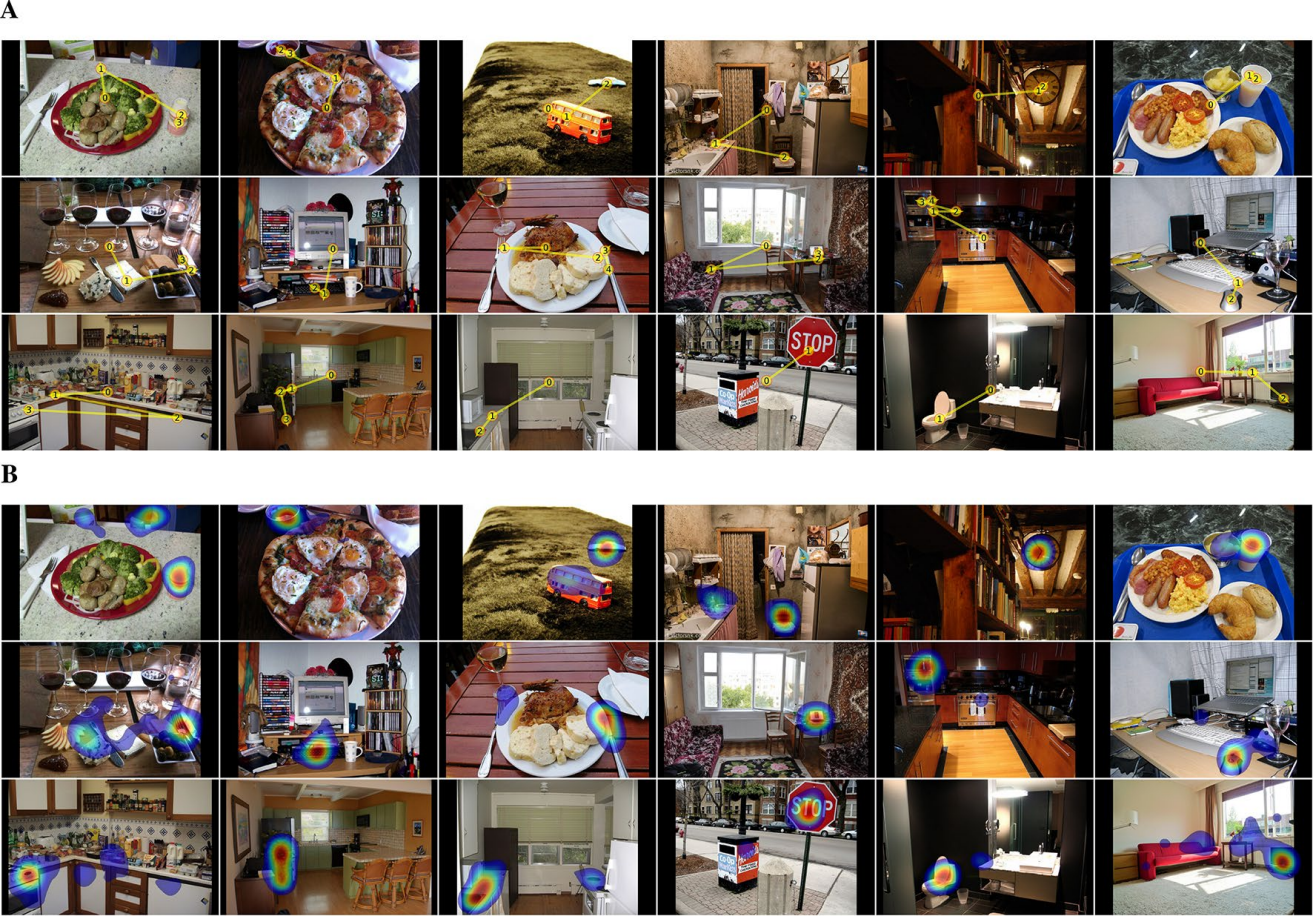
(**A**) Examples of target-present images for each of the 18 target categories. Yellow lines and numbered discs indicate a representative search scanpath from a single participant. From left to right, top to bottom: bottle, bowl, car, chair, (analog) clock, cup, fork, keyboard, knife, laptop, microwave, mouse, oven, potted plant, sink, stop sign, toilet, tv. (**B**) Examples of fixation density maps (excluding initial fixations at the center) computed over participants for the same scenes.

**Figure 2:**
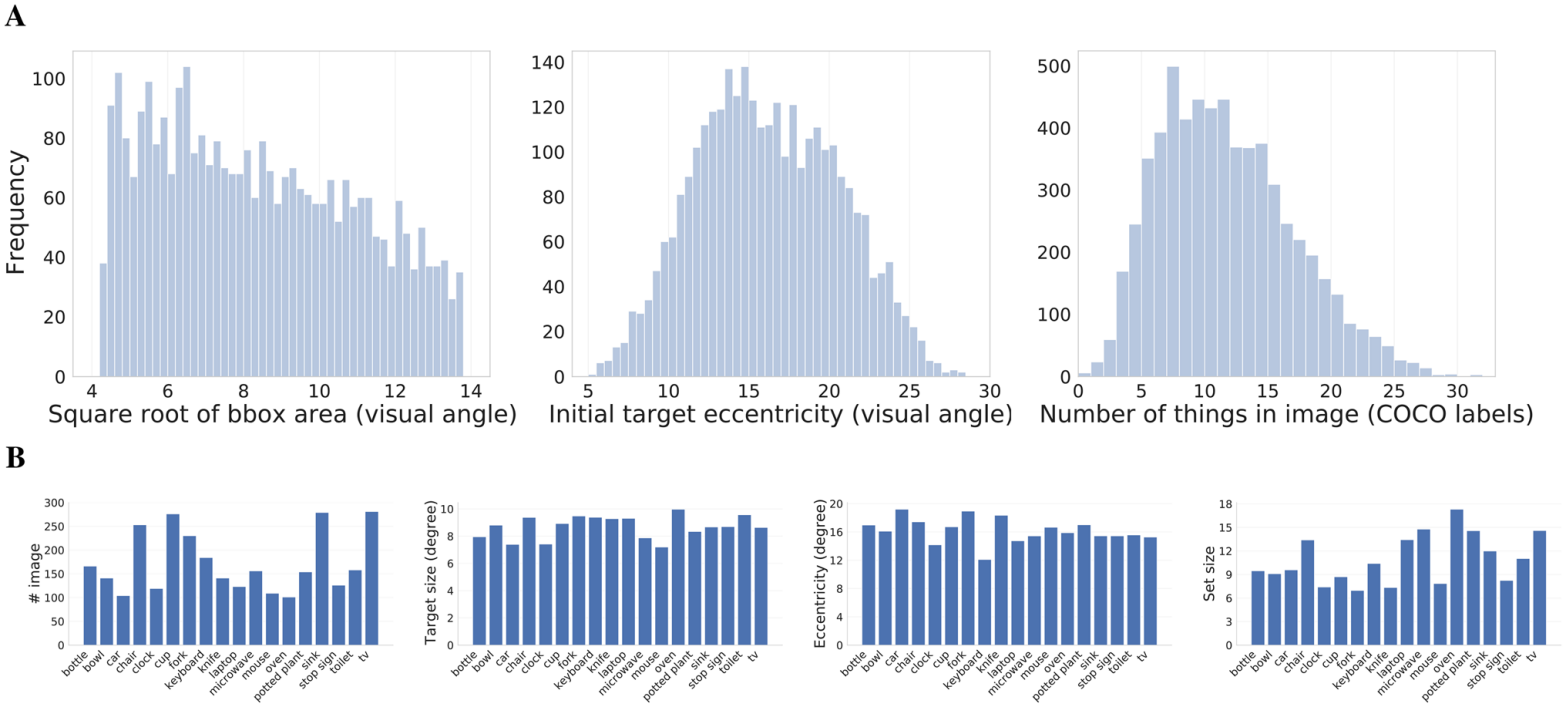
(**A**) Distributions of target sizes, based on the visual angle of their bounding-box areas (left), and initial target eccentricities (middle), both for the target-present images. The number of “things” (objects and “stuff” categories, both based on COCO-stuff labels) appearing in the search images (right). (**B**) Image statistics from COCO-Search18, grouped by the 18 target categories. The left plot shows the number of images, followed by three analyses paralleling those presented in (**A**): averaged target-object size in degrees of visual angle, initial target eccentricity based on bounding-box centers, and the average number of things in an image (a proxy for set size).

**Figure 3:**
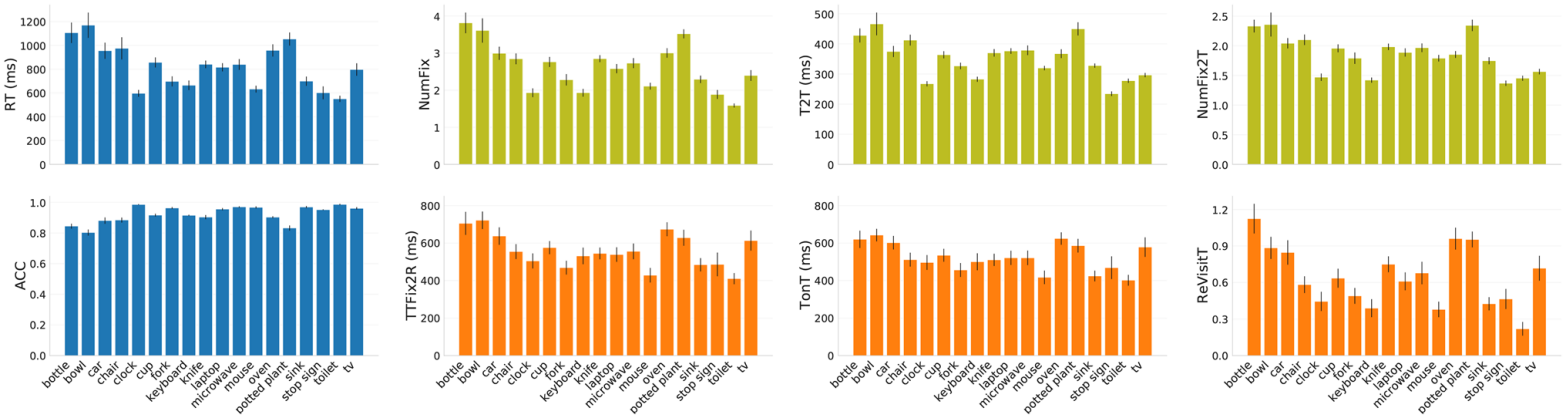
Basic behavioral analyses of the target-present data from COCO-Search18, grouped by the 18 target categories. Blue plots (left two) show the manual measures of reaction time (RT) and response accuracy (ACC). Olive plots (top row) show gaze-based analyses of categorical guidance efficiency: number of fixations made before the button press (NumFix), time until first target fixation (T2T), and number of fixations made until first target fixation (NumFix2T). Orange plots (bottom row) show gaze-based measures of target verification: time from first target fixation until response (TTFix2R), total time spent fixating the target (TonT), and the number of re-fixations on the target (ReVisitT). Values are means over 10 participants, and error bars represent standard errors.

**Figure 4:**
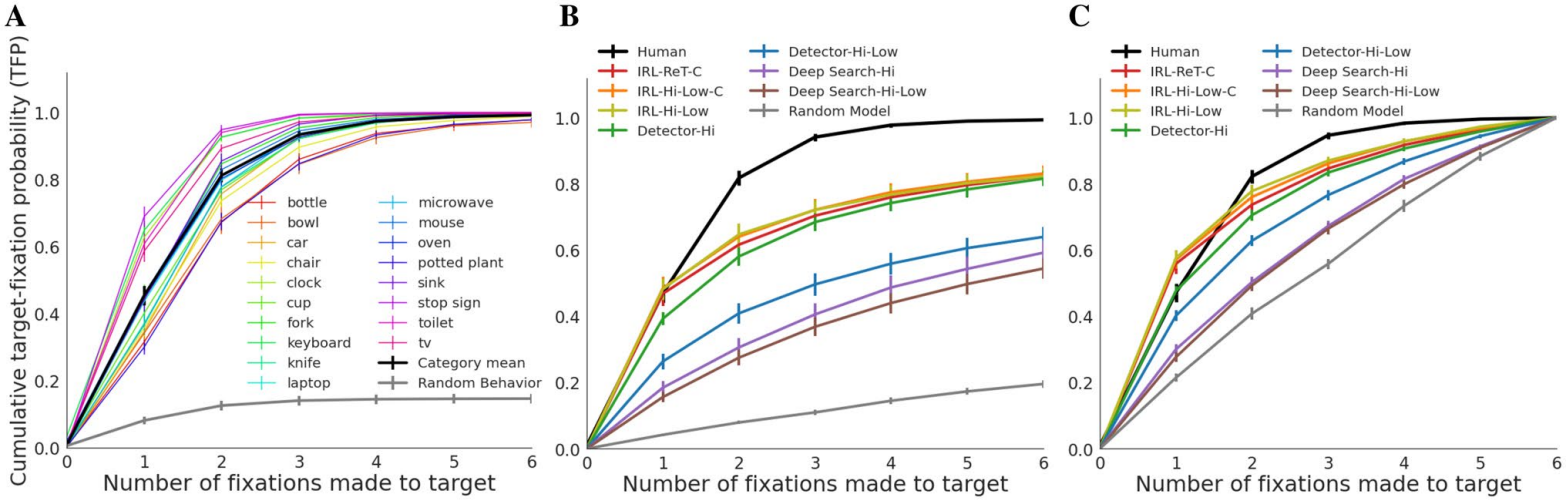
(**A**) Cumulative probability of fixating the target (y-axis; target-fixation probability or TFP) as a function of fixation serial position (x-axis; 0–6), shown individually for the 18 target categories (color lines) and averaged over target types (bold black line). The bottom-most function is a Random Behavior baseline obtained by computing target-fixation probability using a scanpath from the same participant searching for the same target category but in a different image. For the 18 target functions, means were computed by first averaging over images and then over participants, and standard errors were computed over participants. For the averaged behavioral data and the Random Behavior baseline (black and gray lines), means were computed by first averaging over images and then over categories, and standard errors were computed over categories. (**B**) TFP functions generated from model predictions on the test images. Names designate a model type (IRL, Detector, Deep Search) and a state representation (ReT, Hi-Low, Hi, C), separated by hyphens. Average behavioral TFP is again plotted in bold black, this time for just the test data (which explains the small differences from the corresponding function in A, which included the training and testing data). The Random Model baseline was obtained by making six movements of the Hi-Low foveated retina, with ISTs after each, and determining whether any of these movements brought the high-resolution central window to the target. Means were first computed over images and then over categories, and standard errors were computed over categories. (**C**) A re-plot of B, but only including data from trials in which the target was successfully fixated within the first six fixations (i.e., search scanpaths that succeed in locating the target).

## Methods

### Behavioral data collection

COCO-Search18 is built from Microsoft COCO, Common Objects in Context^29^. COCO consists of over 200,000 images of scenes that have been hand-segmented into 80 object categories. This ground-truth labeling of objects in images makes COCO valuable for training computer vision models of object detection^29–33^. However, in order for COCO to be similarly valuable for training models of goal-directed attention, these images would also need to be labeled with the locations fixated by people searching for different target-object goals. COCO-Search18 fills this niche by providing these training labels of search behavior.

The dataset consists of a large-scale annotation of a subset of COCO, 18 of its 80 object categories, with goal-directed search fixations. Participants were 10 Stony Brook University undergraduate and graduate students. All participants provided informed consent in accordance with policies set by the institutional review board at Stony Brook University responsible for overseeing research conducted on human subjects, which also approved the experimental protocol and study methods, all research was performed in accordance with this review board. Each of 10 participants searched for each of 18 target-object categories (blocked) in 6202 COCO images, mostly of indoor scenes. This effort required an average of 12 hours per participant, distributed over 6 days. This substantial behavioral commitment makes it possible to train models of individual searchers^28^, although our focus here is on group behavior. The eye position of each participant was sampled every millisecond using a high-quality eye-tracker under controlled laboratory conditions and procedure, resulting in $\sim$ 70,000,000 gaze-position samples in total. These raw gaze samples were clustered into 299,037 search fixations ($\sim$ 30,000 per participant), which dropped to 268,760 fixations after excluding those from incorrect trials. Figure 1 shows representative images and fixation behavior for each target category. See SM1 for details about: selection criteria (for images, target categories, and fixations), the eye tracker and eye tracking procedure, participant instruction, and a comparison between COCO-Search18 and existing datasets of search behavior.

### Search-relevant image statistics

Figure 2A shows three search-relevant characterizations of the COCO-Search18 images. The left panel shows the distribution of target-object sizes, based on bounding-box COCO labels. This distribution skewed toward smaller targets, with the range constrained by image selection to be between 1% and 10% of the image size (see SM1). The mean visual angle of the targets, based on the square root of bounding-box size, was 8.4$^{\circ }$, about the size of a clenched fist at arm’s length. The middle panel shows the distribution of initial target eccentricities, which is how far the target appeared in peripheral vision, based on center fixation at the start of search. Target eccentricities ranged from 10$^{\circ }$ to 25$^{\circ }$ of visual angle, with a mean of $\sim$ 15$^{\circ }$ eccentricity. The right panel shows the distribution of the number of “things” in each image, again based on the COCO object and stuff labels^34^. Some images depicted only a handful of objects, whereas others depicted 20 or more (keeping in mind that this labeling was coarse). We report this statistic because search efficiency is known to degrade with the number of items in a search array^35^, and a similar relationship has been suggested for the feature and object clutter of scenes^36–38^. Figure 2B again shows these measures, now grouped by the 18 target categories. Target size and initial target eccentricity varied little across target categories, while the measure of set size varied more. See SM2 for analyses showing how each of these three measures correlated with search efficiency, for each target category.

### Search procedure and metrics

The paradigm used for data collection was speeded categorical search^39–41^. The participant’s task was to indicate whether an exemplar of a target category appeared in an image of a scene (Figure S3). They did this by making a target present/absent judgment as quickly as possible while maintaining accuracy. The target category was designated at the start of a block of trials. Half of the search images depicted an exemplar of a target (target-present, TP), and the other half did not (target-absent, TA).

We measure goal-directed attention control as the efficiency in which gaze moves to the search target. Because the target was an object category, the term used for this measure of search efficiency is *categorical target guidance*^40,41^, defined as the controlled direction of gaze to a target-category goal. We consider multiple measures of target guidance in Figure 3, but here we focus on the cumulative probability of fixating the target after each search saccade^42–45^. A target category that can successfully control gaze will be fixated in fewer eye movements compared to one that has less capacity for target guidance. A desirable property of the target-fixation-probability (TFP) function (Figure 4) is that it is meaningful to compute the area under the TFP curve (TFP-auc), which we suggest as a new metric for evaluating search guidance across target categories and models.

### Model comparison

Now that COCO-Search18 exists, what can we do with it? To answer this question we conducted benchmarking to determine how well current state-of-the-art methods, using COCO-Search18, can predict categorical search fixations. To create a context for this model comparison we considered three very different modeling approaches, which all shared a common backbone model architecture, a ResNet50 pre-trained on ImageNet^46^.

Our first approach predicted search fixations using object detectors trained for each of the target categories. We did this by re-training the pre-trained ResNet50 on just the 18 target categories using the COCO labels. Standard data augmentation methods of re-sizing and random crops were used to increase variability in the training samples. We then used these trained detectors to predict search fixations on the test images. For a given target and test image, we obtained a confidence map from the target detector and used it to sample a sequence of fixation locations based on the level of confidence. Note that this approach is pure computer vision, meaning that it uses the image pixels solely and knows nothing about behavior.

With COCO-Search18, however, it is possible to also train on the search behavior. There are multiple ways of doing this. In our second approach we re-trained the same ResNet50, only this time using for labels the fixations made by searchers viewing the training images. Specifically, fixation-density maps (FDMs) were obtained for each TP training image for a given category, and these were used as labels for model training. This model is in a sense a search version of models like DeepGaze II^26^ in the free-viewing fixation-prediction literature, which are also trained to predict FDMs. We therefore refer to this model as Deep Search. Deep Search differs from the Target Detector model in that it is trained on search fixation density to predict search behavior.

For our third modeling approach we used inverse-reinforcement learning (IRL)^47–49^, an imitation-learning method from the machine-learning literature, to simply mimic the search scanpaths observed during training. We chose IRL over other imitation-learning methods because it is based on reward, known to be a powerful driver of behavior^50–52^, but we think it is likely that other imitation-learning methods would perform similarly. The IRL model we used^48^ works by learning, through an adversarial process playing out over many iterations, how to make model-generated behavior, initially random, become more like human behavior. It does this by rewarding behavior that happens to be more human-like. IRL is therefore very different from a Target Detector, but also different from Deep Search, which also gets to use search behavior in its training. The IRL model learns to imitate the search scanpath, meaning the sequence of fixations made to the search target, whereas Deep Search uses only FDMs that do not represent the temporal order of fixations. Because the IRL model used the most search behavior for training, we hypothesized that it would best predict search behavior in our model comparison. See SM3 for additional details about IRL.

### State comparison

In addition to the model comparison, we also compared several state representations used by the models. In the current context, the state is the information that is available to control search behavior, and essential to this are the features extracted from each search image. We refer to the original images as high-resolution (Hi-Res), in reference to the fact that they were not blurred to reflect retina constraints. Extracting features from a Hi-Res image produces a Hi-Res state, and it is this state that is used by most object-detection models in computer vision where the goal is to maximize detection success. Primate vision, however, is profoundly degraded from this Hi-Res state by virtue of the fact that we have a foveated retina. A foveated retina means that high-resolution visual inputs exist only for a small patch of the image at the current fixation location, and blurred everywhere else. Given our goal to model the fixation behavior of the COCO-Search18 searchers, each of whom had a foveated retina, we included this basic biological constraint in the state to determine its importance in model training and prediction of search behavior (see also^53^). Relatedly, and as fundamentally, each new fixation changes the state by allowing high-resolution information to be obtained from the vantage of a new image location. Capturing these fixation-dependent spatio-temporal state changes in the context of search was a core goal in the development of COCO-Search18.

We considered two fovea-inspired states for model training (see^17,28^ for details). In the first we used the method from Perry and Geisler^54^ to compute a Retina-Transformed (ReT) image. A ReT image is a version of the Hi-Res image that is blurred to approximate the gradual loss in visual acuity that occurs when viewing at increasing eccentricities in peripheral vision. Second, we implemented an even more simplified foveated retina consisting of just a high-resolution central patch (7$^{\circ }$ $\times$ 7$^{\circ }$ visual angle) surrounded by low-resolution “peripheral” vision elsewhere, with the critical difference from the ReT image being that only a single level of blur (Gaussian filter with $\sigma$ = 2) was used to approximate the low-resolution periphery. Computing the gradual blur used in the ReT image was computationally very demanding, and the inclusion of the simpler Hi-Low state was motivated largely to reduce these computational demands (ReT requires $\sim$ 15$\times$ the processing time per image). However, having this condition also enabled a needed initial evaluation of how veridically low-level visual-system constraints need to be followed when training deep-network models of human goal-directed behavior.

We also considered two spatio-temporal state representations for how information is accumulated with each new fixation in a search scanpath. A behavioral consequence of having a foveated retina is that we make saccadic eye movements, and the order in which these eye movements are made correspond to different visual states. Our first spatio-temporal state assumed a high-resolution foveal window that simply moves within a blurred image. This means that each change in fixation brings peripherally blurred visual inputs into clearer view, and causes previously clear visual inputs to become blurred. This spatio-temporal state representation is aligned most closely with the neuroanatomy of the oculomotor system, so we will consider this to be the default state. However, this default state representation assumes that foveally-obtained information on fixation *n* is completely lost by fixation *n+1*, and indeed something like this is true for high-resolution information about visual detail^55^. However, this state fails to capture any memory for the fixated objects that persists over eye movement, which is also known to exist^56^. To address the potential for an object context to build over fixations, we therefore also used a state that accumulates the high-resolution foveal views obtained at each fixation in the search scanpath, a state we refer to as Cumulative (-C). Over the course of multi-fixation search, the Hi-Low-C state would therefore accumulate high-resolution foveal snapshots with each new fixation, progressively de-blurring what would be an initially moderately-blurred version of the image. We explore these two extremes of information preservation during search so as to inform future uses of a fovea-inspired spatio-temporal state representation to train deep network models.

### General model methods

All of the models followed the same general pipeline. Each 1050 $\times$ 1680 image input was resized to 320 $\times$ 512 pixels to reduce computation. This is what we refer to as the Hi-Res image (or just Hi); the ReT and Hi-Low images were computed from this. These images were passed through the ResNet50 backbone to obtain 20 $\times$ 32 feature map outputs, with the features extracted from these images now reflecting either Hi-Res, ReT, or Hi-Low states, respectively. Different models were trained using these features and others, as described in the Model Comparison section, and all model evaluations were based on a 70% training, 10% validation, and 20% testing, random split of COCO-Search18 within each target category. See SM3 for additional details about the training and testing separation, and Figure  S15 for how the two compare on search performance measures.

From the trained models we obtain *priority maps*, a term we use instead of *saliency maps* to make clear the top-down origin of the attention bias, and then use these maps to predict the search fixations in test images. The priority map for the Target Detector was a map of detector confidence values at each pixel location, and fixations were sampled probabilistically from this confidence map. The priority map for Deep Search is its prediction of the FDM, given the input image and the model’s learned mapping between image features and the FDM ground-truth during training. The priority map for the IRL model is the reward map recovered during its training, which recall occurred during its learning to mimic search behavior. Because this search behavior was itself reward driven, the priority map for the IRL model is therefore a map of the total reward expected by making a sequence of search fixations to different locations in a test image. The IRL model was additionally constrained to have an action space discretized into a 20 $\times$ 32 grid, which again was done to reduce computation time. A given action, here a change in fixation, is therefore a selection of 1 from 640 possible grid cells, a sort of limitation imposed on the spatial resolution of the model’s oculomotor system. The selected cell was then mapped back into 320 $\times$ 512 image space by upscaling, and the center of this cell became the location of the model’s next fixation. The non-IRL models made their action selection directly in the 320 $\times$ 512 image space, with higher priority values selected with higher probability.

All of the model $\times$ state combinations in our comparison were required to make six changes in fixation for each test image. This number was informed by the behavioral data showing that the probability of target fixation was clearly at ceiling by the sixth eye movement (Figure 4A). To produce these 6-fixation scanpaths, we iterated the fixation generation procedure using inhibitory spatial tagging (IST), which is a mechanism serving the dual functions of (1) breaking current fixation, thereby enabling gaze to move elsewhere, and (2) discouraging the refixation of previously searched locations. IST has long been used by computational models of free viewing^57,58^ and search^59,60^. Here we enforce IST by setting the priority map to zero after each fixation over a region having a radius of 2.5$^{\circ }$ visual angle (based on a 3 $\times$ 3 grid within the 20 $\times$ 32 action space). IST was applied identically after each fixation made by all of the models. This was true even for models that did not have a foveated retina, such as a Target Detector with a Hi-Res state, in which case IST was applied to the image locations selected for “fixation”. See SM3 for additional details.

The nomenclature that we adopted for the model comparison consists of the model type as the base and the state representation as a suffix. If the spatio-temporal state is cumulative, there is a second suffix of -C. For example, the IRL-ReT-C model accumulates graded-resolution foveal views of an image with each reward-driven eye movement. Although our aim is to explore as systematically as possible each state for every model, for some models a given state representation is not applicable. For example, it makes no sense for the IRL model to use the Hi-Res state. Because that state representation does not change from one search fixation to the next it would be impossible to learn fixation-dependent changes in state, thereby defeating the purpose of using the IRL method. Similarly, it makes no sense to have a cumulative state for anything but the IRL model, as the others would be unable to use this information. However, it does make sense to test a Target Detector and Deep Search on a Hi-Low state as well as a Hi-Res state, and these models are included in the Table 1 model evaluation.

**Table 1:** Results from fixation-prediction models (rows) using multiple scanpath metrics (columns) applied to the COCO-Search18 test images.

	TFP-AUC $\varvec{\uparrow }$	Probability mismatch $\varvec{\downarrow }$	Scanpath ratio $\varvec{\uparrow }$	Sequence score $\varvec{\uparrow }$	Multimatch $\varvec{\uparrow }$			
					shape	direction	length	position
Human	5.200	–	0.862	0.489	0.903	0.736	0.880	0.910
Random model	0.744	4.455	0.392	0.266	0.832	0.579	0.783	0.755
Detector-Hi	4.001	1.209	0.680	0.411	0.877	0.665	0.837	0.872
Detector-Hi-Low	2.975	2.225	0.601	0.370	0.863	0.640	0.820	0.833
Deep Search-Hi	2.519	2.681	0.579	0.348	**0.890**	0.627	**0.867**	0.861
Deep Search-Hi-Low	2.282	2.918	0.546	0.333	0.882	0.617	0.859	0.848
IRL-ReT-C	4.170	1.131	0.731	0.418	0.879	0.673	0.842	0.874
IRL-Hi-Low-C	**4.262**	**1.031**	0.747	**0.419**	0.886	**0.677**	0.849	**0.885**
IRL-Hi-Low	4.245	1.036	**0.753**	0.417	0.884	**0.677**	0.847	**0.885**

Arrows indicate the direction of better prediction success, and values in bold indicate best predictions across the model comparison. In the case of Sequence Score and MultiMatch, “Human” refers to an oracle method whereby one searcher’s scanpath is used to predict another searcher’s scanpath; “Human” for all other metrics refers to observed behavior. See the main text for additional details about the scanpath-comparison metrics, and SM3 for purely spatial comparisons using the AUC, NSS, and CC metrics.

**Figure 5:**
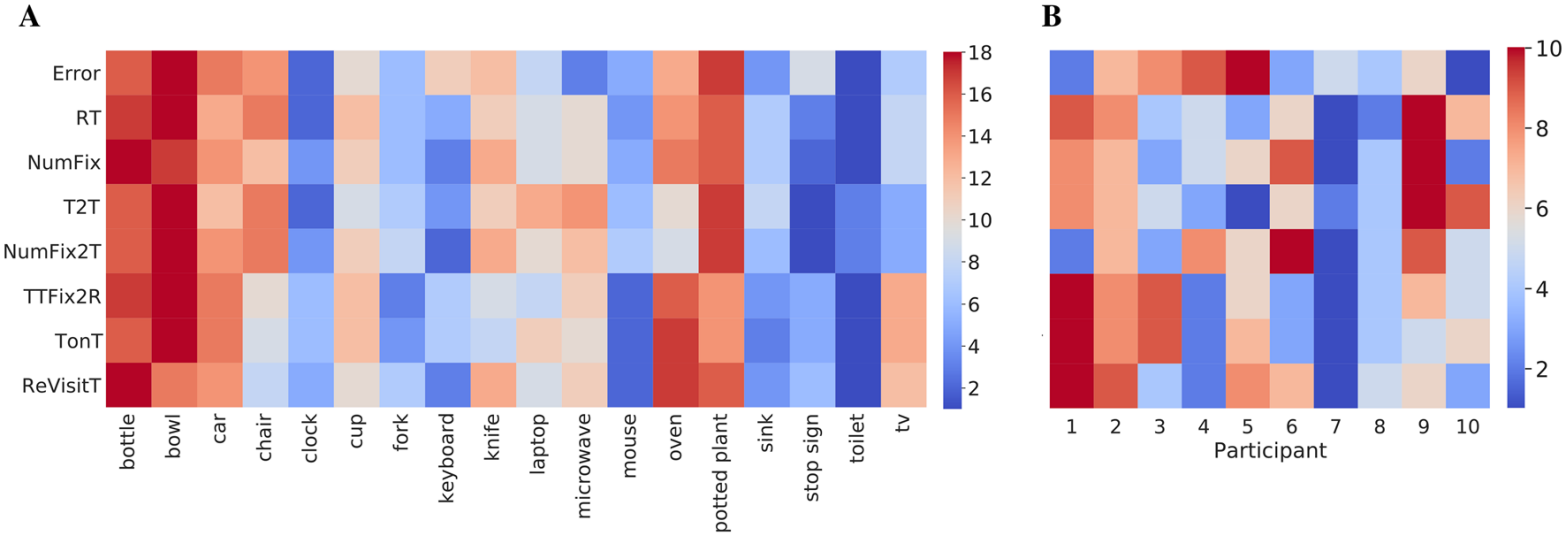
(**A**) Ranked target-category search efficiency [1–18], averaging over participants. Redder color indicates higher rank and harder search targets, bluer color indicates lower rank and easier search. Target category is grouped (columns) and shown for multiple performance measures (rows). These measures include: response Error, reaction time (RT), number of fixations (NumFix), time to target (T2T), number of fixations to target (NumFix2T), time from first target fixation until response (TTFix2R), time spent fixating the target (TonT), and the number of target re-fixations (ReVisitT). (**B**) A similar ranking of the target-present data, only now for participant efficiency (columns 1–10), averaged over target category. Performance measures and color coding are the same as in panel (**A**).

## Results

### Behavioral performance

We interrogated COCO-Search18 using multiple performance measures. Figure 3 reports these analyses for each of the target categories. Analyses can be conceptually grouped into manual measures (accuracy and response time; blue plots), gaze-based measures of categorical guidance (number of fixations before the button press, and both the time and number of fixations until the first target fixation; olive plots), and measures of target verification time (time from first target fixation until the button press, total time spent fixating the target, and the number of target re-fixations; orange plots). What is clear from these analyses is that, except for accuracy, there is wide variability across target categories in these measures, and this variability creates fertile ground for future model development. Also clear from Figure 3 is that there is considerable correlation among some of these measures, perhaps most evident among the search guidance measures where the shapes of the plots look similar. We include these different measures, not to suggest their independence, but rather as a courtesy to readers who may be familiar with different measures.

Figure 5 is a matrix visualization of these analyses, now with color coding a ranking of search efficiency. In Figure 5A, the deepest red for each measure (row) indicates the least efficient (or most difficult) search over the 18 target categories, and the deepest blue indicates the most efficient (or easiest) search. The appearance of columns in this visualization captures the agreement among the measures. More subtle patterns in the data can also be seen. For example, the two predominately red columns at the left indicate agreement in that the bottle and bowl objects were difficult targets, speculatively because these target categories have particularly high variability in their image exemplars. Relatedly, appearing near the right are two of the consistently easiest targets, stop signs and toilets, both having relatively well-defined category membership. Figure 5B shows a similar plot, only now performance is averaged over target categories and plotted for individual participants. Search accuracy and efficiency clearly differ among the participants in this ranking. Participants 7 and 8 were better searchers than Participants 2 and 9, meaning that they tended to find the target faster and with fewer fixations while keeping a low error rate. Differences in search strategy can also be seen from this visualization. Participant 1 searched the display carefully, resulting in few missed targets, but this person’s search was not very efficient. In contrast, Participant 4 was quick to find and verify targets, but had relatively low accuracy. See Table S1 for detailed individual behavioral data and see SM2 for parallel analyses of the target-absent data from COCO-Search18.

However, arguably the gold-standard measure of attention control is the cumulative probability of fixating the target after each saccade made during search, the target-fixation probability (TFP). Figure 4A shows TFP functions for the first six search saccades, averaged over participants and plotted for individual target categories. The mean behavior over targets is indicated by the bold black function. The noteworthy pattern is that the slope of the group function is far steeper than that of the chance baseline, obtained by computing TFP using a scanpath from a different image but from the same participant and target category. On average, about half of the targets were fixated with the very first saccade. By the second saccade TFP jumped to 0.82, and by the third saccade it reached a performance ceiling of 0.94, which increased only slightly after saccades 4–6. This high degree of attention control means that, although we aimed to create a search dataset having a moderate level of difficulty, COCO-Search18 skews easy. This is due in part to unexpectedly large practice effects (see SM2 for details). However, it is fortuitous that such a strong attention-control signal exists in the behavioral data, given the challenge faced by even start-of-the-art models in predicting this simple search behavior.

### Model evaluation

Fixation prediction models broadly fall into two groups, models that predict the spatial distribution of locations fixated by participants viewing an image (i.e., the FDM), and models that predict both the location and order of the fixations made by a person viewing an image (i.e., the scanpaths). In a search task, fixation behavior changes dramatically over the first few eye movements^61^, making it important to consider the spatio-temporal fixation order. For this reason, we will focus on spatio-temporal fixation prediction here and defer discussion of purely spatial FDM prediction to SM4, and especially Table S2. Both types of prediction were based on the 6-saccade sequences that each model was required to make for each test image. Specifically, 10 6-fixation scanpaths (excluding the initial fixation) were predicted for each test image by sampling probabilistically from the generated priority map, and for each of these search scanpaths the model behavior was analyzed up to first fixation on the target, or six changes in fixation, whichever came first.

Predictions of the spatio-temporal order of search fixations are usually made with respect to the efficiency in finding a search target. For example, predicting the probability of the target being fixated by the first search saccade, the second, etc. These target-based predictions capture the efficiency of search, where the goal is to find the target, and models making this type of prediction have been the more common in the search literature^45,62^. Here we use three metrics to evaluate the success of these predictions. Two of these metrics were derived from the TFP function (Figure 4A): TFP-AUC, which is the area under the cumulative target-fixation-probability curve, and Probability Mismatch, which sums over each fixation in a scanpath the absolute differences between the behavioral and model TFP. The third metric, Scanpath Ratio, is the Euclidean distance between the initial fixation location (roughly the center of the image) and the location of the target (center of bounding box) divided by the summed Euclidean distances between the fixation locations in the search scanpath^43^. It is a search efficiency metric because an initial saccade that lands directly on the target would yield a Scanpath Ratio of 1, and all less efficient searches would be < 1.

An alternative to predicting target guidance over the spatio-temporal search scanpath is to predict the scanpath itself. This approach assumes that any target guidance would be reflected in the sequence of fixated image locations leading up to the target decision. We considered two metrics for comparing behavioral and predicted search scanpaths: Sequence Score, which clusters scanpaths into strings and uses a string matching algorithm for comparison^63^, and MultiMatch, which takes a multi-dimensional approach to computing scanpath similarity^64,65^. Both metrics capture properties of the spatio-temporal search scanpath and place less importance on the fact that there is a search target. SM4 should be consulted for additional details about these metrics.

Table 1 provides an evaluation of how each model$\times$state combination fared in fair comparison using these metrics. As we hypothesized, the three IRL models generally outperformed the others (see Table S3 for statistical tests). They did so for every metric except MultiMatch, where all the models performed similarly. The only other model that was comparably predictive was Detector-Hi, but this model has no fovea and is therefore the least biologically plausible. A perhaps clearer picture of this model comparison can be obtained by comparing the behavioral TFP function to ones computed for each model. Figure 4B shows this evaluation of search efficiency for each of the model$\times$state combinations (in color) and for the mean search behavior (in black), limited to the TP test data. Focusing first on state comparisons, we did not find large differences between the states tested. Whether blur was graded or binary appeared not to matter, as indicated by the very similar TFP functions for the ReT and the Hi-Low states using the IRL model. This pattern also appeared in Table 1, where the IRL models differed by tiny margins. For this reason, and its far greater computational efficiency, we adopted only the Hi-Low state in the other model comparisons (therefore, there are no Deep Search-ReT or Detector-ReT models). Similarly, but specific to the IRL model, it made little difference whether or not the state accumulated high-resolution visual information with each fixation in a search scanpath. The fact that the IRL model seemed not to use this accumulated visual information is broadly consistent with the view that very little high-resolution information is preserved across saccades^55^. However, it did matter whether the state included a foveated retina or not, as exemplified by the difference between Hi-Res and Hi-Low states for the Detector model. This state comparison suggests that future work may want to avoid manipulations of fine-grained retinal blur and assumptions about intersaccadic visual memory, and focus on adding more basic limitations on human visual perception to a model’s pipeline, with the inclusion of a Hi-Low foveated retina being one example.

All of the tested models made reasonable predictions of search behavior in this challenging benchmark, where “reasonable” is liberally defined as bearing greater resemblance to the human behavior than the chance baseline. However, the Deep Search models and the Detector-Hi-Low model were clearly less efficient in their search behavior than either human behavior or any of the IRL models. This poor relative performance is likely caused by these models not capturing the serial order of search fixations, and that this order matters. A corollary finding is that the IRL models, because they learned these spatio-temporal sequences of search fixations, better predicted search behavior. This was true for all the IRL models, which all predicted the efficiency of the first search fixation almost perfectly (IRL models vs. Human at fixation 1 with post-hoc t-tests, all $\textit{p}s_{bonferroni}$ = 1.0). Also interesting is the degree that an object detector (Detector-Hi) can predict search behavior, supporting previous speculation^66^. If an application’s goal is to predict a person’s early fixation behavior during search without regard for biological plausibility, a simple object detector will work well based on our testing with COCO-Search18. Another finding from Figure 4B is that none of the models achieved the high level of successful target fixation exhibited in human performance. Performance ceilings after six saccades (termed *fixated-in-6 accuracy*) ranged from 0.54 (Deep Search-Hi-Low) to 0.83 (IRL-Hi-Low-C), all well below the near perfect fixated-in-6 accuracy (0.99) from human searchers (post-hoc t-tests with all $\textit{p}s_{bonferroni}$ < 0.001). These lower performance plateaus, undoubtedly reflecting limitations in current object detection methods, means that the models tended either to fixate the target efficiently in the first one or two eye movements (like people), or tended not to fixate the target at all (unlike people). If a model cannot represent the features used for target guidance as robustly as people, there may be images for which there is essentially no guidance signal, and on these inefficient search trials the number of eye movements needed to fixate the target would often be greater than six, hence the performance plateaus.

These different performance ceilings are problematic in that they conflate limitations arising from object detection with limitations in effective target prioritization, as measured by search efficiency. For example, a strength of the TFP-AUC metric is that it is grounded in the TFP functions from Figure 4B, but this means that it includes the different performance ceilings in its measure and this weakens it as a pure measure of attention control. To address this concern, in Figure 4C we again plot TFP functions, but now only for trials in which the target was successfully fixated within the first six saccades. By restricting analysis to only trials having perfect fixated-in-6 accuracy, the metric becomes more focused on search efficiency. By this measure, and keeping in mind that the data are now skewed toward easier searches, the IRL-Hi-Low-C and IRL-Hi-Low models remain the most predictive overall, although now all IRL models overestimate slightly the efficiency of the first search saccade. But perhaps the biggest winner in this comparison is the Detector-Hi model, which now predicts TFP almost perfectly after the first fixation, and has generally improved performance for subsequent fixations. We tentatively conclude that simple prioritization of fixations by an object detector predicts reasonably well the prioritization of behavioral fixations in visual search. The losers in this comparison were the Deep Search models, which remained less efficient than human behavior even after normalization for fixated-in-6 accuracy.

## Discussion

Recent years taught us the importance of large datasets for model prediction, and this importance extends to models of attention control. COCO-Search18 is currently the largest dataset of goal-directed search fixations, having sufficient number to be used as labels for training deep network models. We conducted a systematic (but still incomplete) exploration of models and state representations to provide some initial context for the types of model predictions that are possible using COCO-Search18, given current state-of-the-art (or nearly so). This model comparison focused on the degree that search behavior was used during training, ranging from none (Detector), to some (Deep Search), to entire search-fixation scanpaths (IRL). With respect to the IRL model, its use with COCO-Search18 is the first attempt to predict the spatio-temporal movements of goal-directed attention by training on human search behavior. We found that the IRL model was far more predictive of search efficiency than the Detector-Hi-Low model or either of the Deep Search models, despite the Deep Search models using methods considered to be state-of-the-art in the fixation-prediction literature on free-viewing behavior. In our state comparison we focused on the different ways that a primate foveated retina, and its movement, might be represented and used to train fixation prediction models. We also extensively benchmarked COCO-Search18, both in terms of the search behavior that it elicited, analyzed using multiple behavioral measures and metrics, and in terms of the predictive success of models ranging in their degree of training on the COCO-Search18 behavior. All this means that COCO-Search18 can be used immediately to start generating new testable hypotheses. But likely the greatest contribution of this work is yet to come. With a dataset the size and quality of COCO-Search18, opportunities exist to explore new policies and reward functions for predicting goal-directed control that have never before been possible^28^. Our hope is that COCO-Search18 will strengthen the bridge that human attention has built between the machine learning and behavioral science literatures.

COCO-Search18 is now part of the MIT/Tuebingen Saliency Benchmark, previously the MIT Saliency Benchmark but renamed to reflect the group that is now managing the competition. The training, validation, and test images in COCO-Search18 are already freely available as part of COCO^29^. Researchers are also free to see and use COCO-Search18’s training and validation search fixations, but the fixations on the test images are withheld. As part of a managed benchmark, in a separate track it will be possible to upload predictions and have them evaluated on this test dataset. We invite you to participate in this good-natured adversarial competition, and we hope that you enjoy using COCO-Search18: https://github.com/cvlab-stonybrook/Scanpath_Prediction.

## Supplementary Information

Supplementary Information 1.

## Data Availability

The COCO-Search18 dataset described in the current paper is available at: https://github.com/cvlab-stonybrook/Scanpath_Prediction.
