# Supplementary material for: COCO-Search18 fixation dataset for predicting goal-directed attention control
Source: Sci Rep. 2021 Apr 22;11:8776. doi: 10.1038/s41598-021-87715-9 (PMC8062491; doi:10.1038/s41598-021-87715-9)
Supplement: Supplementary file 1 — Supplementary Information 1. [file 41598_2021_87715_MOESM1_ESM.pdf]

COCO-Search18: A Dataset for Predicting Goal-directed Attention Control

Yupei Chen[1], Zhibo Yang[2], Seoyoung Ahn[1], Dimitris Samaras[2], Minh Hoai[2], Gregory Zelinsky[1, 2]
Department of Psychology, Stony Brook University[1]
Department of Computer Science, Stony Brook University[2]

Supplementary Materials

SM1: Behavioral Data Collection

Comparable datasets of search behavior

Figure S1 shows how COCO-Search18 compares to other large-scale datasets of search behavior. To our knowledge, there were only three such image datasets that were annotated with human search fixations[8, 10, 23]. In terms of number of fixations, number of target categories, and number of images, COCO-Search18 is far larger. The PET dataset[10] collected search fixations for six animal target categories in 4,135 images selected from the Pascal VOC 2012 dataset[9], but the search task was non-standard in that participants were asked to “find all the animals” rather than search for a particular target category. This paradigm is therefore search at the super-ordinate categorical level, which is far more weakly guided than basic-level search[16]. Gaze fixations were also recorded for only 2 seconds/image, and multiple targets often appeared in each scene. The microwave-clock search dataset (MCS[23]) is our own work and a predecessor of COCO-Search18. In collecting data for the 18 target categories in COCO-Search18 we had to start somewhere, and our first two categories were microwaves and clocks (although the datasets differed for even those two categories due to the use of different exclusion criteria). Until recently, perhaps the best dataset of search fixations was from[8], but it is relatively small, limited to only the search for people in scenes, and is now a decade old. Note that, whereas there are larger datasets with respect to free-viewing fixations (SALICON[13]) or fixations collected using other visual tasks (POET[19]), these tasks were not visual search and therefore these datasets cannot be used to train models of search behavior. These collective inadequacies demanded the creation of a newer, larger, and higher-quality dataset of search fixations, enabling deep network models to be trained on people’s movements of attention as they pursue target-object goals.

Selection of target categories and search images

Here we more fully describe how we selected from COCO’s trainval dataset[15] the 18 target categories and the 6,202 images included in COCO-Search18. A goal in implementing our selection criteria was to elicit the behavior that we are trying to measure, namely, the guidance of search fixations by a target category. We also put care into excluding images that might elicit other gaze patterns that would introduce noise with respect to identifying the target-control signal. This sort of attention to detail is uncommon in datasets created for the training of deep network models, where the approach seems to be "the more images the better". But whereas this is usually true because more images leads to better-trained models, in creating a dataset of human behavior this more-is-better impulse should be tempered with some quality control to be confident that the behavior is of the purported type. In the current context this behavior should be search fixations that are guided to the target, because search fixations that are unguided have less value as training labels. Because a standard search paradigm collects behavioral responses for both TP and TA images, separate selection criteria were needed. All image selection was based on object labels and/or bounding boxes provided by COCO. On this point, while inspecting the images that were ultimately selected we noticed that exemplars in some categories were mislabeled, probably due to poor rater agreement on that category. For instance, several chair exemplars were mislabeled as couches, and vice versa. Rather than attempting to correct these mislabels, which would be altering COCO, we decided to keep them and tolerate a higher-than-normal error rate for the affected categories. This action seemed best, given our plan to discard error trials from the search performance analyses in our study, but researchers interested in interpreting button press errors in COCO-Search18 should be aware of this labeling issue.

Target-present image selection. Six criteria were imposed on the selection of images to be used for target-present search trials.

(1) Images were excluded if they depicted people or animals. We did this to avoid the known biases to fixate on these objects when they appear in a scene[4, 14]. Such biases would compete with guidance from target-category features, thereby distorting study of the target-bias that is more central to search.
(2) Images were excluded if they depicted multiple instances of the target. A scene showing a classroom with many chairs would therefore be excluded from the “chair” target category because one, and only one, instance of a chair would be allowed in an image.
(3) Images were excluded if the size of the target, measured by the area of its bounding box, was smaller than 1% or larger than 10% of the total image area. This was done to create searches that were not too hard or too easy.
(4) Images were excluded if the target appeared at the image center, based on a 5×5 grid. We did this because the participant’s gaze was pre-positioned at this central location at the start of each search trial.
(5) Images were excluded if their width/height ratio fell outside the range of 1.2-2.0 (based on a screen ratio of 1.6). This criterion excluded very elongated images, which we thought might distort normal viewing behavior.
(6) Images, and entire image categories, were excluded if the above criteria left fewer than 100 images per object category. We did this because fewer than 100 images would likely be insufficient for training and testing a deep network model specific to that object category.

Applying these exclusion criteria left 32 object categories from COCO's original 80. Given that this left still far too many images for people to practically annotate with search fixations, we decided to attempt exclusion of images where targets were highly occluded or otherwise difficult to recognize. We did this out of concern that such images would largely introduce noise into the search behavior. To do this, we trained object detectors on cropped views of these 32 categories, and excluded images if the object bounding boxes had a classification confidence $< .99$. Specifically, for these 32 categories we created a validation set consisting of images meeting the selection criteria and a training set consisting of the images that did not. The bounding box of the object, for each of the 32 object classes, was then cropped in the image to obtain the positive training samples. Negative samples were same-sized image patches that had 25% intersection with the target (area of intersection divided by area of target), meaning that they were class-specific hard negatives. All cropped patches (over 1 million) were resized to 224×224 pixels while maintaining the aspect ratio using padding. The classifier was a ResNet50 pre-trained on ImageNet, which we fine-tuned by dilating the last fully-connected layer and re-training on 33 outputs (32+"Negative"). Images were excluded if the cropped object patch had a classification score of less than .99. This procedure resulted in 18 categories with at least 100 images in each category, totaling 3,131 TP images.

Two final exclusion criteria were implemented by manual selection. First, for the clock target category we included only images of analog clocks, meaning that we excluded digital clocks from being clock targets. We did this because the features of analog and digital clocks are highly distinct and very different, and we were concerned that this would introduce variability in the search behavior and reduce data quality. Five images depicting only digital clocks were excluded for this reason. Lastly, images from all 18 of the target categories were screened for objectionable content, which we defined as offensive content or content evoking discomfort or disgust. The "toilet" category had the most images (17) excluded for objectionable content, with a total of 25 images excluded across all target categories. After implementing all exclusion criteria discussed in this section, we obtained 3,101 TP images from 18 categories: bottle, bowl, car, chair, (analog) clock, cup, fork, keyboard, knife, laptop, microwave, (computer) mouse, oven, potted plant, sink, stop sign, toilet, and tv. See Figure 2 for the specific number of images in each category.

Target-absent image selection. To balance the selection of the 3,101 TP images, we selected an equal number of TA images from COCO. To do this, we kept the criteria excluding images depicting people or animals, extreme width/height image ratios, and images with objectionable content, all as described for the TP image selection, but added two more exclusion criteria that were specific to each of the 18 target-object categories.

(1) Images were excluded if they depicted an instance of the target, a prerequisite for a TA image.

(2) Images were excluded if they depicted less than two instances of the target category's siblings, a criterion introduced to discourage searchers from making TA responses purely on the basis of scene type. For example, a person might be biased to make a TA response if they are searching for a toilet target and the image is a street scene. Because COCO has a hierarchical organization, parent, child, and sibling relationships can be used for image selection. For example, COCO defines the siblings of a microwave to be an oven, toaster, refrigerator, and sink, all under the parent category of appliance. By requiring that the TA scenes for a target category have at least two of that category's siblings, we impose a sort of scene constraint that minimizes target-scene inconsistency and makes a scene appropriate to use as a TA image. A scene that has an oven and a refrigerator is very likely to be a kitchen, thereby making it difficult to answer on the basis of scene type alone whether a microwave target is present or absent.

These exclusion criteria still left us with many thousands more TA images than we needed, so we sampled randomly within each of the 18 target categories to match the 3,101 TP images.

Order of target-category presentation

Collecting the search behavior for 6,202 images required dividing each participant's effort into six days of testing. Each testing session was conducted on a different day, lasted about 2 hours, and consisted of about 1000 search trials, evenly divided between TP and TA. Because images from different categories can overlap (e.g., images depicting a microwave may also depict an oven), the presentation order of the target-category blocks was constrained to minimize the repetition of images in consecutive categories and consecutive sessions. For example, because 49 images satisfied the selection criteria for both the sink and microwave target categories, we prevented the microwave and sink categories from appearing in, not only the same session, but the sessions preceding and following. We did this to minimize possible biases resulting from seeing the same scene in different search contexts. A heuristic for maximizing this distance between repeating images resulted in the following fixed target category presentation order across the six sessions:

(1) tv + sink;

(2) fork + chair;

(3) car + bowl + potted plant + mouse;

(4) knife + keyboard + oven + clock;

(5) cup + laptop + toilet;

(6) bottle + stop sign + microwave.

Each participant viewed from Session 1 to Session 6, or from Session 6 to Session 1, with this order counterbalanced across participants.

Data-collection procedure

Participants were 10 Stony Brook University undergraduate and graduate students, 6 males and 4 females, with ages ranging from 18–30 years. All had normal or corrected to normal vision, by self report, were naive with respect to task design and paradigm when recruited, and were compensated with course credit or money for their participation. Informed consent was obtained from each participant at the beginning of testing, in accordance with the Institutional Review Board responsible for overseeing human-subjects research at Stony Brook University.

The target category was designated to participants at the start of each block. This was done using the type of display shown in Figure S2 for the potted-plant and analog clock categories. The name of the target category was shown in text at the top, with examples of objects that would, or would not, qualify as exemplars of the named category. In selecting exemplars to illustrate as positive target-category members, we attempted to capture key categorical distinctions at a level immediately subordinate to the target category. When needed, we also gave negative examples by placing a red X through the object. We did this to minimize potential confusions and to enable the participant to better define the target category's boundary.

The procedure (Figure S3) on each trial began with a fixation dot appearing at the center of the screen. To start a trial, the participant would press the "X" button on a game-pad controller while carefully looking at the fixation dot. An image of a scene would then be displayed and the participant's task would be to answer, "yes" or "no", whether an exemplar of the target category appears in the displayed scene by pressing the right or left triggers of the game-pad, respectively. The search scene remained visible until the manual response. Participants were told that there were an equal number of TP and TA trials, and that they should make their responses as fast as possible while maintaining high accuracy. No accuracy or response time feedback was provided.

The presentation of images during the experiment was controlled by Experiment Builder (SR research Ltd., Ottawa, Ontario, Canada). Stimuli were presented to participants on a 22-inch LCD monitor (1680$\times$1050 pixel resolution) at a viewing distance of 47cm from the monitor, enforced by chin and head rests. These viewing conditions resulting in horizontal and vertical visual angles of $54^\circ \times 35^\circ$, respectively. Participants were asked to keep their gaze on the fixation point at the start of each trial, but were told that they should feel free to move their eyes as they searched. Eye movements were recorded throughout the experiment using an EyeLink 1000 eye-tracker in tower-mount configuration (SR research Ltd., Ottawa, Ontario, Canada). Eye-tracker calibrations occurred before every block or whenever necessary, and these 9-point calibrations were not accepted unless the average calibration error was $\leq .51^\circ$ and the maximal error was $\leq .94^\circ$. The experiment was conducted in a quiet laboratory room under dim lighting conditions.

SM2: Behavioral evaluation of COCO-Search18

Effects of set size and target eccentricity

The visual search literature has done excellent work in identifying many of the factors that increase search difficulty (for reviews, see:[6,7,21,22]). Larger set sizes (number of items in the search display), smaller target size, larger target eccentricity, and greater target-distractor similarity are all known to make search more difficult. However, most of this work was done in the context of simple stimuli, and generalization to realistic images is challenging. For example, what to consider an object in a scene is often unclear, making it difficult to define a set size[18]. Objects in images also do not usually come annotated with labels and bounding boxes. These problems of object segmentation and identification, which largely do not exist for search studies using object arrays, become significant obstacles to research when scaled up to images of scenes.

With COCO-Search18, we can begin to ask how the search for targets in images is affected by set size and target eccentricity. Set size is determined based on the COCO object and stuff labels, which collectively map every pixel in an image to an object or stuff category. Set size is the count of the number of these labels for a given image. Figure S4 shows the relationship between the number of fixations made on an image, averaged over participants, and the set size of that image, grouped by target category. Some target categories, such as laptop, oven, microwave, and potted-plant, have significant positive set size effects (r = .21 to .37, ps $\leq$ .01), indicating a less efficient search with more objects. A similar pattern is shown in Figure S5 for the relationship between the number of fixations on a search image and the initial visual eccentricity of the target (distance between the image center and the target bounding-box center), where for these same objects there was a decrease in search efficiency with increasing target eccentricity. For other target object categories, such as: stop sign, fork, and keyboard, search efficiency was unaffected by either set size or target eccentricity (ps $>$ .05), possibly because these objects are either highly salient (stop sign) or highly constrained by scene context (keyboard).

Distance between search fixations and the target

How much closer does each search fixation bring gaze to the target? We analyzed this measure of search efficiency and report the results in Figure S6. Plotted is the Euclidean distance between the target location and the locations of the starting fixation (0) and the fixation locations after the first six eye movements (1-6). The most salient pattern is the rapid decrease in fixation-target distance in the first two new fixations, which dovetails perfectly with the steep increase in the cumulative probability of target fixation over these same eye movements reported in Figure 4A. From a starting location near the center of the image, these eye movements brought gaze steadily closer to the target. Note that because this fixation-target distance is averaged over images and participants, the roughly 5 degrees of visual angle at the bottom of these functions should not be misinterpreted as gaze being this distance from the target on a given trial. More interpretable

are the overall trends, where a steep drop in distance is followed by a plateau, or even a smaller increase in distance with the 5th and 6th new fixations. This small increase is likely an artifact of these 5 and 6-fixation trials being the most difficult, with more idiosyncratic search behavior.

Target-absent search fixations

In the main text we focused on the TP data, where the guidance signal is clearer and the modeling goals are better defined, but we conducted largely parallel analyses of the TA data. Figure S7A shows representative TA images with fixation data from one participant, and Figure S7B shows FDMs from all participants for the same images. Comparing these data with the TP data from Figure 1, it is clear that people made many more fixations in the absence of a target. This was expected from the search literature, but it should also be noted that the FDMs are still much sparser than what would be hypothesized by an exhaustive search. Paralleling Figure 3, in Figure S8 we report applicable analyses of the TA search behavior. These are grouped by manual accuracy and response time, and the mean number of fixations made before the target-absent button press terminating a trial. Note that accuracy was high (low false positive error rate) for all of the target categories except chairs and cups, with the reason for the former already discussed in the context of mislabeling and the reason for the latter likely reflecting an occasionally challenging category distinction (e.g., some bottles can look like some cups). Also note that there was an average of only five fixations made during search, even on the TA search trials. As in Figure 5, Figure S9 visualizes the agreement and other patterns among these measures. The rows show ranked performance, with dark red indicating more difficult (or least efficient) search and dark blue indicating relatively easy or efficient search. The columns in Figure S9A group the measures by target category. Similar to the TP data, there was again good consistency among the measures. Also consistent is the fact that bottles and cups were among the most difficult target categories, whereas the toilet category was the easiest. There was also evidence in the TA data for a speed-accuracy trade-off for some target categories. For example, microwaves and stop signs had relatively low error rates, but these categories were searched with relatively high effort, as measured by ranked response time and number of fixations. Figure S9B visualizes the measures by participant instead of category, where we again found individual differences between participants in search efficiency.

Practice effects

Each of the participants contributing to COCO-Search18 searched more than 6000 images, making it possible to analyze how their search efficiency improved with practice. Figure S10 shows practice effects for both response time (top) and the number of fixations before the button press (bottom), where we define practice effects as performance on the first 1/3 of the trials compared to performance on the last 1/3 of the trials for each target category. Practice effects were larger for TA trials (right) than for TP trials (left), noting the differences in y-axes scales, and that considerable differences existed across categories. Some categories, such as bottles, showed large practice effects, while other categories, such as analog clocks, showed none at all. We speculate that this difference is due to some categories requiring more exemplars to fully learn compared to others. For example, analog clock was perhaps the most well defined of COCO-Search18's categories, and bottle certainly one of the least well defined, creating greater opportunity to better learn the bottle category with practice over trials.

Search fixation durations

Figures S11 and S12 show density histograms of the search fixation durations for the TP and TA data, respectively, plotted for each of the target categories. Fixation durations are plotted across the x-axes with a bin size of 50ms, and y-axes show the normalized probability density at each fixation. Of note in the TP data is that the mode initial fixation durations (blue lines) were a bit longer than the mode duration of the rest (averaged mode difference = 63ms), consistent with the very strong guidance observed in the initial eye movements, and they tended to have more bi-modal distributions. The main peak was at $\sim$250 ms, with a smaller and very short-latency peak at $\sim$50 ms that is likely a truncation artifact of fixation duration being measured relative to the onset of the search display. In contrast, the distributions of second fixations (orange lines) were consistently shorter, even relative to the subsequent fixations. Speculatively, this may be due to a greater proportion of the first new fixations being "off object"[24], which are often followed by short-latency corrective saccades that bring gaze accurately to an object. This interpretation is consistent with the high probability of the target being fixated by the second eye movement (Figure 4A). As for the subsequent fixations, they tended to be short ($\sim$200ms) and not highly variable in their durations. The TA fixations showed similar trends, except for the durations of the second fixations no longer differing from the rest.

Saccade amplitudes

We also analyzed the distribution of saccade amplitudes during visual search, defined here as the Euclidean distance between consecutive fixations in visual angle. Figure S13 and Figure S14 show the distributions of saccade amplitudes in the TP and TA data, respectively. In the TP data, saccade amplitudes were larger in some categories (toilet and stop sign) than others (bottle and potted plant), likely because easier target categories could be identified from farther in the visual periphery. There was also evidence for bimodality in the amplitude distributions, shown most clearly for clocks, forks, stop signs, and tvs. We speculate that this bimodality reflects larger-amplitude exploratory saccades mixed with smaller-amplitude saccades used in the verification of an object category. Mean saccade amplitudes in the TA data were clearly larger than for the TP data ($t(17) = 11.79$, $p < .001$), and this difference was consistent across target categories (all

ps ≤ .001). We attribute this to the relatively large viewing angle of the search displays (54 × 35 degrees of visual angle) creating a greater need for exploration, but this is also speculation. The distributions of saccade amplitudes were also more consistent across categories in the TA data, with there being weaker evidence of bi-modality.

SM3: Model Methods

Training and testing datasets

Model success depends on the training dataset being an accurate reflection of the test dataset. When the training dataset includes a behavioral annotation, as does COCO-Search18, it is therefore important to know that similar patterns exist in the training and testing search behavior. The analyses shown in Figure 5A included images from all of COCO-Search18, which recall were randomly split into 70% for training, 10% for validation, and 20% for testing. Figure S15 replots the data from Figure 5A, but divides it into the training/validation (left) and testing (right) datasets. Note the high agreement between the testing and train/val datasets across this battery of behavioral performance measures.

Inverse Reinforcement Learning

The specific inverse-reinforcement learning (IRL) method that we used was generative adversarial imitation learning (GAIL[12]) with proximal policy optimization (PPO)[20]. The model policy is a generator that aims to create state-action pairs that are similar to human behavior. The reward function (the logarithm of the discriminator output) maps a state-action pair to a numeric value. The generator and discriminator are trained within an adversarial optimization framework to obtain the policy and reward functions. The discriminator's task is to distinguish whether a state-action pair was generated by a person (real) or by the generator (fake), with the generator aiming to fool the discriminator by maximizing the similarity between its state-action pairs and those from people. The reward function and policy that are learned from the fixation-annotated images during training are then used to predict new search fixations in the unseen test images.

SM4: Performance metrics and model evaluation

Metrics for comparing search efficiency and scanpaths

We considered five metrics for quantifying search efficiency and comparing search scanpaths (Table 1). Two metrics for quantifying search efficiency follow directly from the group target-fixation probability (TFP) function shown in Figure 4. The first of these computes the area under the TFP curve, a metric we refer to as TFP-auc. Second, and relatedly, we compute the sum of the absolute differences between the human and model target-fixation-probabilities in a metric that we refer to as Probability Mismatch. A third metric for quantifying overt search efficiency is Scanpath Ratio. It is the Euclidean distance between the initial fixation location and the target divided by the summed Euclidean distances between the fixation locations in the search scanpath[11]. It is an efficiency metric because an initial saccade that lands directly on the target would give a Scanpath Ratio of 1, meaning that the distance between starting fixation and the target would be the same as the summed saccade distance. These three metrics emphasize target-fixation efficiency by penalizing either the number of fixations or the saccade-distance traveled to achieve the target goal. The final two metrics focus on scanpath comparison, and specifically comparing the search scanpaths between people and the models. The first of these scanpath-comparison metrics computes a Sequence Score by first converting a scanpath into a string of fixation cluster IDs, and then using a string matching algorithm[17] to measure the similarity between the two strings. Figure S16 shows examples of behavioral and model scanpaths and their sequence scores to develop an intuition for this metric. Lastly, we use MultiMatch[1,5] to measure the scanpath similarity at the pixel level. MultiMatch measures five aspects of scanpath similarity: shape, direction, length, position, and duration. We excluded the duration measure from our use of this metric because the models in our comparison group did not predict fixation duration. See Table S3 for the results of statistical tests comparing predictions from each pair of models.

Comparing predicted and behavioral fixation-density maps (FDMs)

Search has a temporal dynamic, making a metric for capturing the spatio-temporal sequence of fixations preferred over ones that compare only FDMs, where this temporal component is disregarded. However, the prediction of FDMs is common for free-viewing tasks, and because there is no technical reason why FDM metrics cannot be applied to search we do so here in the hope that the visual saliency literature finds this comparison useful. Models generated scanpaths having a maximum length of 6 new fixations, but FDMs were constructed only from those fixations leading up to the first fixation on the target, just as FDMs were constructed from the behavioral fixations. We used three widely accepted metrics for comparing predicted against observed FDMs. Area Under the Receiver Operating Characteristic Curve (AUC) uses a predicted priority map as a binary classifier to discriminate behavioral fixation locations from non-fixated locations. Normalized Scanpath Saliency (NSS) finds the model predictions at each of the behavioral fixation locations, then averages and normalizes these values. Lastly we computed a Pearson's Correlation Coefficient (CC) between the predicted and behavioral FDMs, although this metric reflects only the degree of linear relationship between predicted and behavioral FDMs (for additional discussion, see: Borji & Itti[2]; Bylinskii et al.[3]). Table S2 reports the results of an evaluation comparing model predictions of search FDMs to behavioral search FDMs using each of these metrics. The findings that we report in the main text in the context of scanpath prediction also hold in the case of FDM prediction. Specifically, the IRL-Hi-Low-C model outperformed the others, and did so for all three metrics. Additionally, the Detector-Hi model also performed relatively well in all the metrics, supporting our conclusion that a simple detector does a relatively good job in predicting fixations in

visual search.

References

1. Nicola C Anderson, Fraser Anderson, Alan Kingstone, and Walter F Bischof. A comparison of scanpath comparison methods. *Behavior research methods*, 47(4):1377–1392, 2015.
2. Ali Borji and Laurent Itti. State-of-the-art in visual attention modeling. *PAMI*, 35(1):185–207, 2012.
3. Zoya Bylinskii, Tilke Judd, Aude Oliva, Antonio Torralba, and Frédo Durand. What do different evaluation metrics tell us about saliency models? *IEEE transactions on pattern analysis and machine intelligence*, 41(3):740–757, 2018.
4. Moran Cerf, Jonathan Harel, Wolfgang Einhäuser, and Christof Koch. Predicting human gaze using low-level saliency combined with face detection. In *Advances in neural information processing systems*, pages 241–248, 2008.
5. Richard Dewhurst, Marcus Nyström, Halszka Jarodzka, Tom Foulsham, Roger Johansson, and Kenneth Holmqvist. It depends on how you look at it: Scanpath comparison in multiple dimensions with multimatch, a vector-based approach. *Behavior research methods*, 44(4):1079–1100, 2012.
6. John Duncan and Glyn W Humphreys. Visual search and stimulus similarity. *Psychological review*, 96(3):433, 1989.
7. Miguel P Eckstein. Visual search: A retrospective. *Journal of vision*, 11(5):14–14, 2011.
8. Krista A Ehinger, Barbara Hidalgo-Sotelo, Antonio Torralba, and Aude Oliva. Modelling search for people in 900 scenes: A combined source model of eye guidance. *Visual cognition*, 17(6-7):945–978, 2009.
9. M. Everingham, L. Van Gool, C. K. I. Williams, J. Winn, and A. Zisserman. The PASCAL Visual Object Classes Challenge 2012 (VOC2012) Results. http://host.robots.ox.ac.uk/pascal/VOC/index.html.
10. Syed Omer Gilani, Ramanathan Subramanian, Yan Yan, David Melcher, Nicu Sebe, and Stefan Winkler. Pet: An eye-tracking dataset for animal-centric pascal object classes. In *2015 IEEE International Conference on Multimedia and Expo (ICME)*, pages 1–6. IEEE, 2015.
11. John M Henderson, Phillip A Weeks Jr, and Andrew Hollingworth. The effects of semantic consistency on eye movements during complex scene viewing. *Journal of experimental psychology: Human perception and performance*, 25(1):210, 1999.
12. Jonathan Ho and Stefano Ermon. Generative adversarial imitation learning. In *Advances in Neural Information Processing Systems*, pages 4565–4573, 2016.
13. Ming Jiang, Shengsheng Huang, Juanyong Duan, and Qi Zhao. Salicon: Saliency in context. In *The IEEE Conference on Computer Vision and Pattern Recognition (CVPR)*, June 2015.
14. Tilke Judd, Krista Ehinger, Frédo Durand, and Antonio Torralba. Learning to predict where humans look. In *2009 IEEE 12th international conference on computer vision*, pages 2106–2113. IEEE, 2009.
15. Tsung-Yi Lin, Michael Maire, Serge Belongie, James Hays, Pietro Perona, Deva Ramanan, Piotr Dollár, and C Lawrence Zitnick. Microsoft coco: Common objects in context. In *European conference on computer vision*, pages 740–755. Springer, 2014.
16. Justin T Maxfield and Gregory J Zelinsky. Searching through the hierarchy: How level of target categorization affects visual search. *Visual Cognition*, 20(10):1153–1163, 2012.
17. Saul B Needleman and Christian D Wunsch. A general method applicable to the search for similarities in the amino acid sequence of two proteins. *Journal of molecular biology*, 48(3):443–453, 1970.
18. Mark B Neider and Gregory J Zelinsky. Exploring set size effects in scenes: Identifying the objects of search. *Visual Cognition*, 16(1):1–10, 2008.
19. Dim P Papadopoulos, Alasdair DF Clarke, Frank Keller, and Vittorio Ferrari. Training object class detectors from eye tracking data. In *European conference on computer vision*, pages 361–376. Springer, 2014.
20. John Schulman, Filip Wolski, Prafulla Dhariwal, Alec Radford, and Oleg Klimov. Proximal policy optimization algorithms. *arXiv:1707.06347*, 2017.
21. Anne Treisman and Stephen Gormican. Feature analysis in early vision: evidence from search asymmetries. *Psychological Review*, 95(1):15, 1988.
22. Gregory Zelinsky. A theory of eye movements during target acquisition. *Psychological review*, 115(4):787, 2008.
23. Gregory Zelinsky, Zhibo Yang, Lihan Huang, Yupei Chen, Seoyoung Ahn, Zijun Wei, Hossein Adeli, Dimitris Samaras, and Minh Hoai. Benchmarking gaze prediction for categorical visual search. In *Proceedings of the IEEE Conference on Computer Vision and Pattern Recognition Workshops*, pages 0–0, 2019.
24. Gregory J Zelinsky. Tam: Explaining off-object fixations and central fixation tendencies as effects of population averaging during search. *Visual Cognition*, 20(4-5):515–545, 2012.

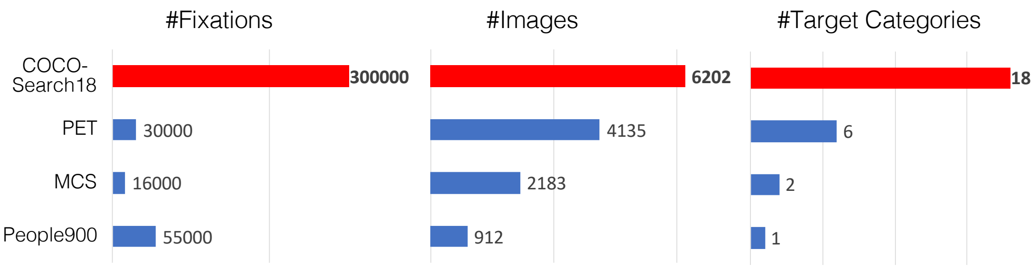

Figure S1. Comparisons between COCO-Search18 and other large-scale datasets of search behavior. COCO-Search18 is the largest in terms of number of fixations (∼300,000), number of target categories (18), and number of images (6,202).

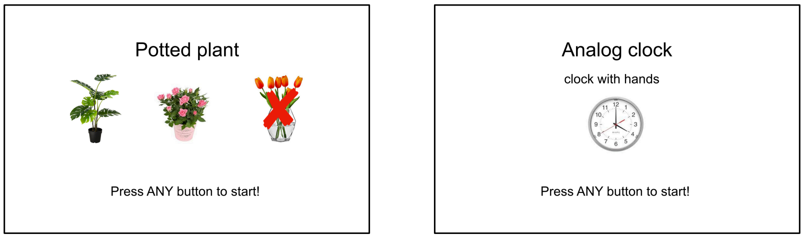

Figure S2. Examples of target-designation displays, shown for the potted-plant and analog clock targets, that preceded the block of trials for a given target category.

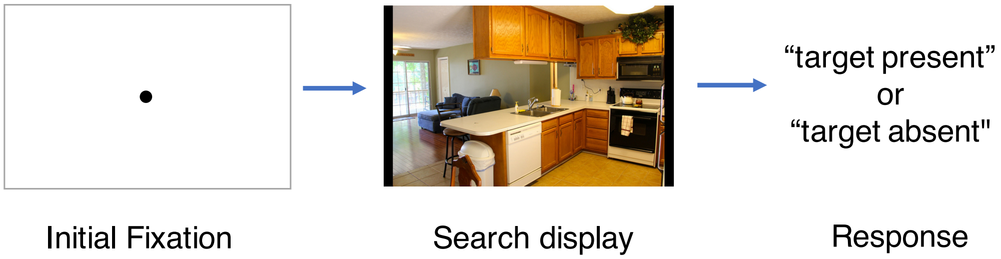

Figure S3. Example of the search procedure. Each trial began with a fixation dot appearing at the center of the screen. Participants would start a trial by pressing a button on a game-pad controller while carefully looking at the fixation dot. An image of a scene would then be displayed and the participant's task was to make a speeded "yes" or "no" target-presence judgment by pressing the right or left triggers, respectively, of a game-pad controller.

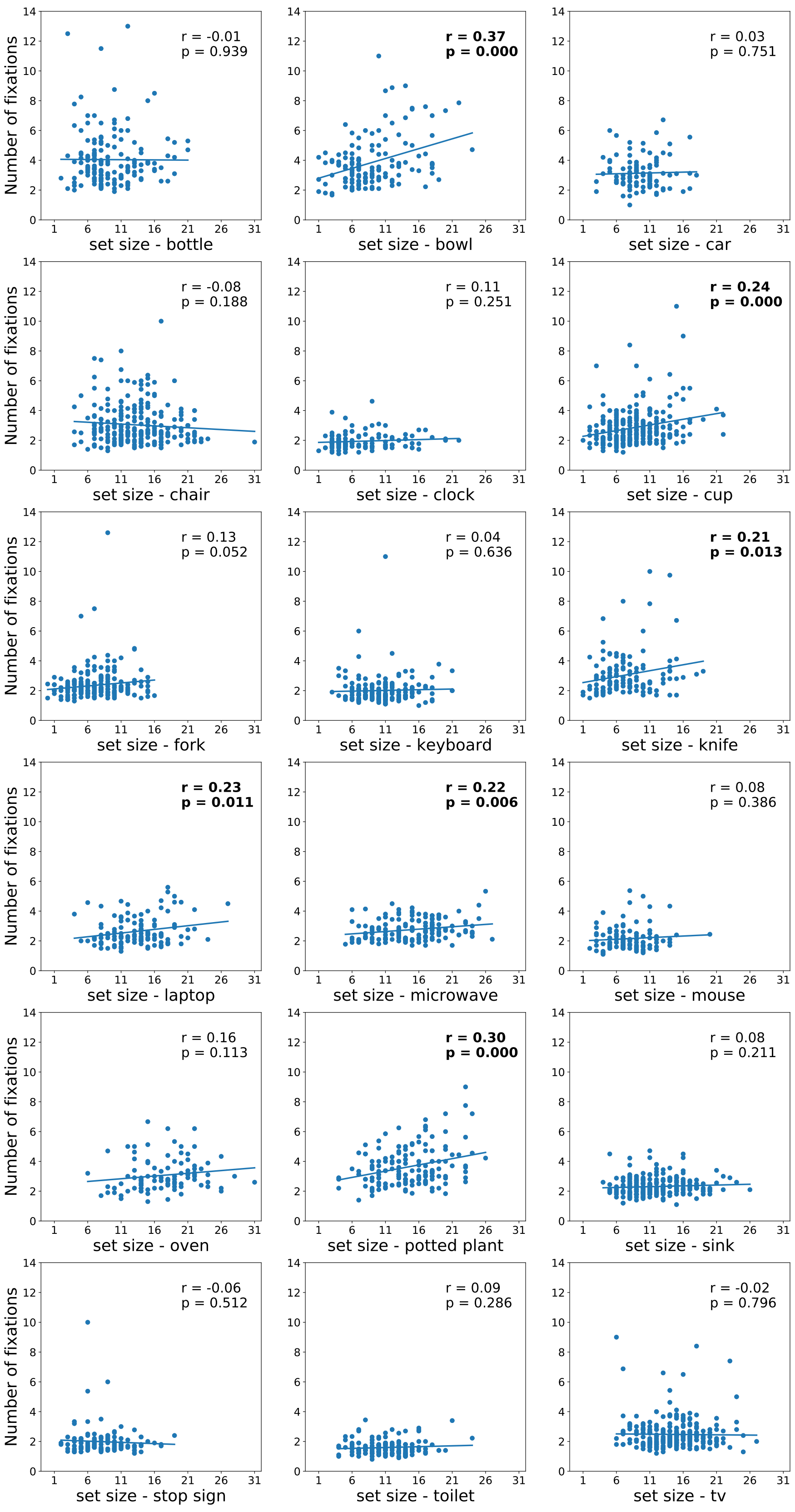

Figure S4. Number of fixations made on the target-present images plotted as a function of the set sizes of those images (using COCO object and stuff labels), averaged over participants and grouped by target category.

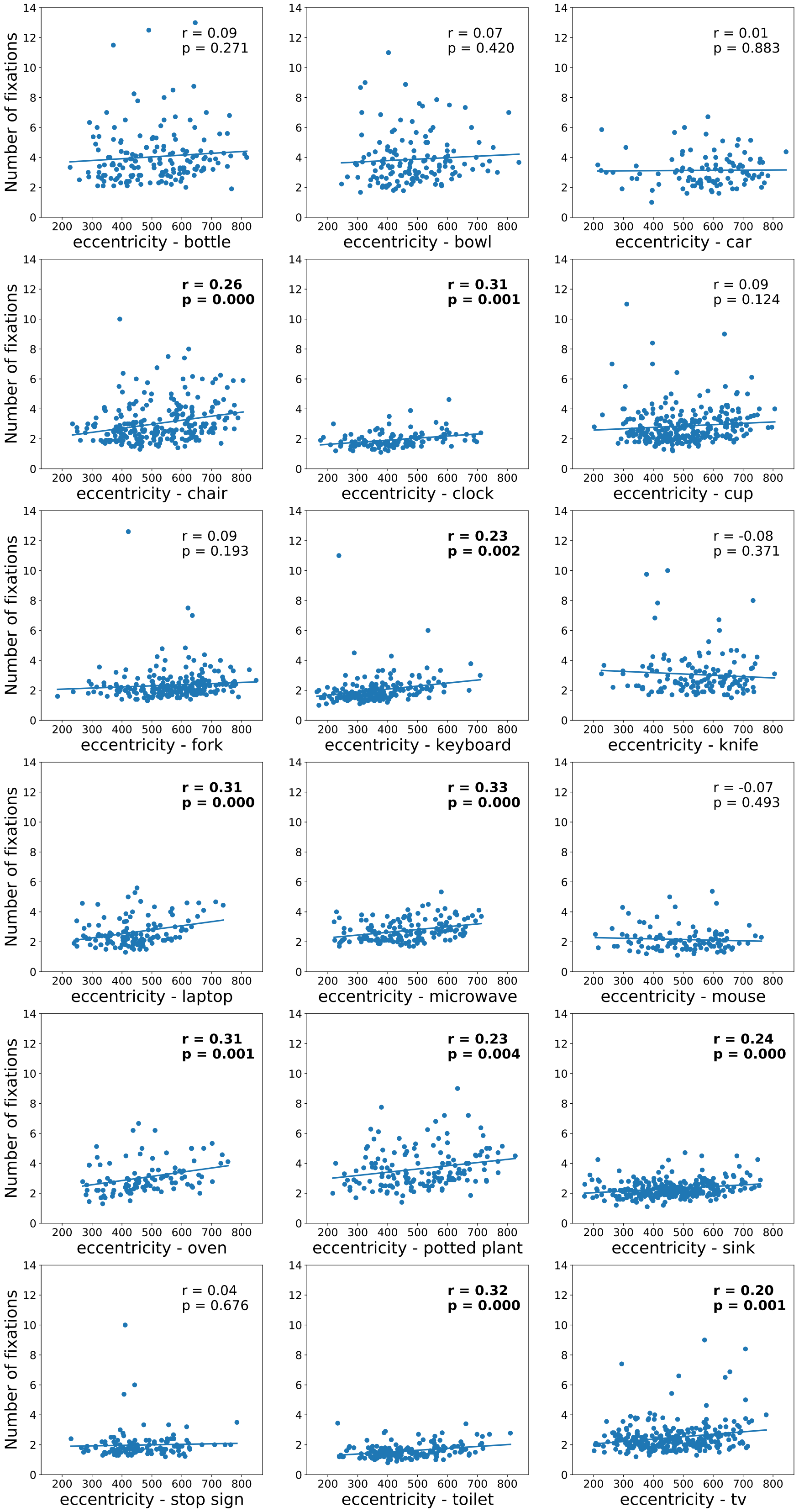

Figure S5. Number of fixations made on the target-present images plotted as a function of initial target eccentricity (using the center of the COCO bounding-box), averaged over participants and grouped by target category.

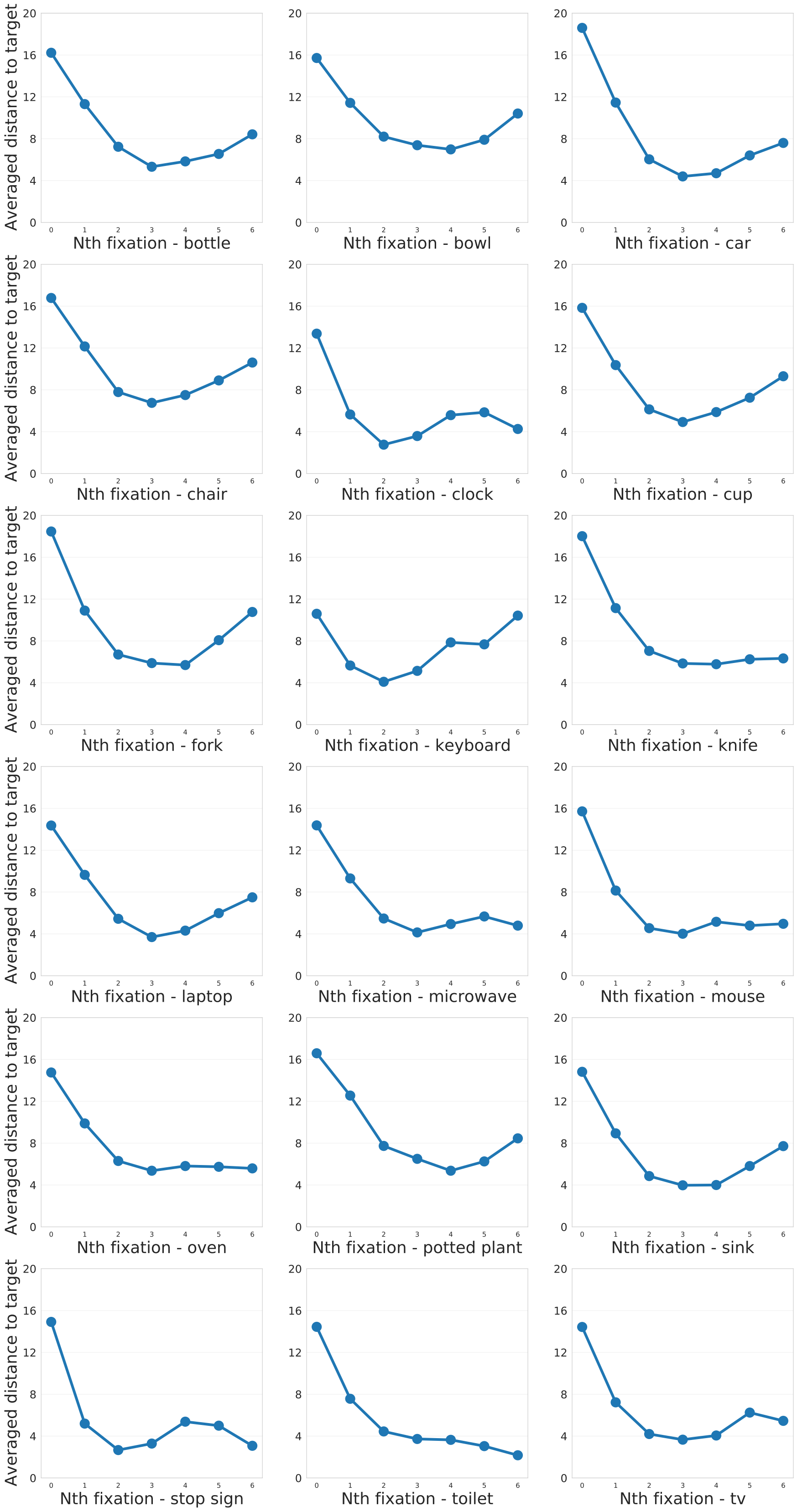

Figure S6. Averaged Euclidean distance (in visual angle) between gaze and the target's center (using COCO bounding-box labels) over the first 6 saccades, grouped by target category.

A

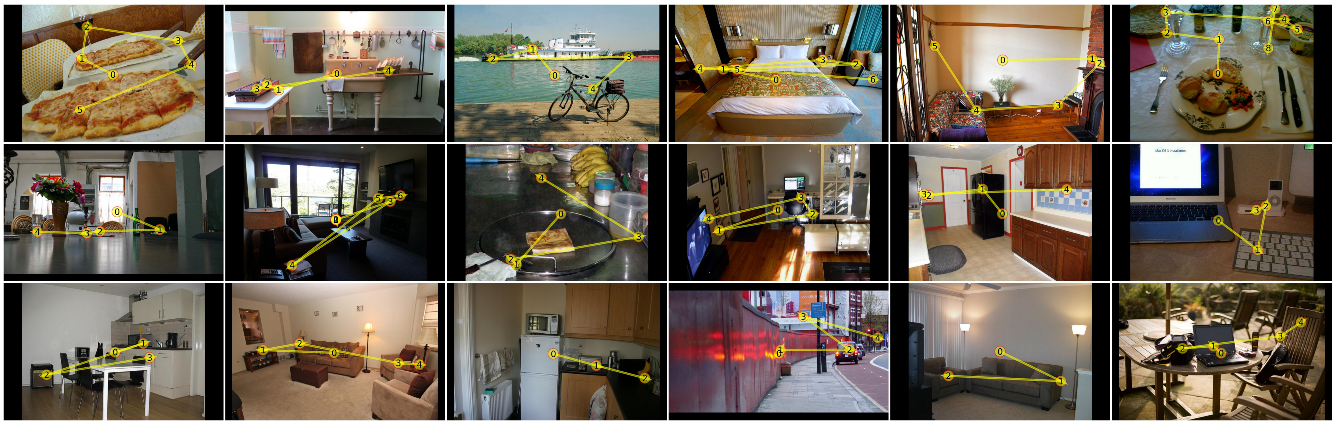

B

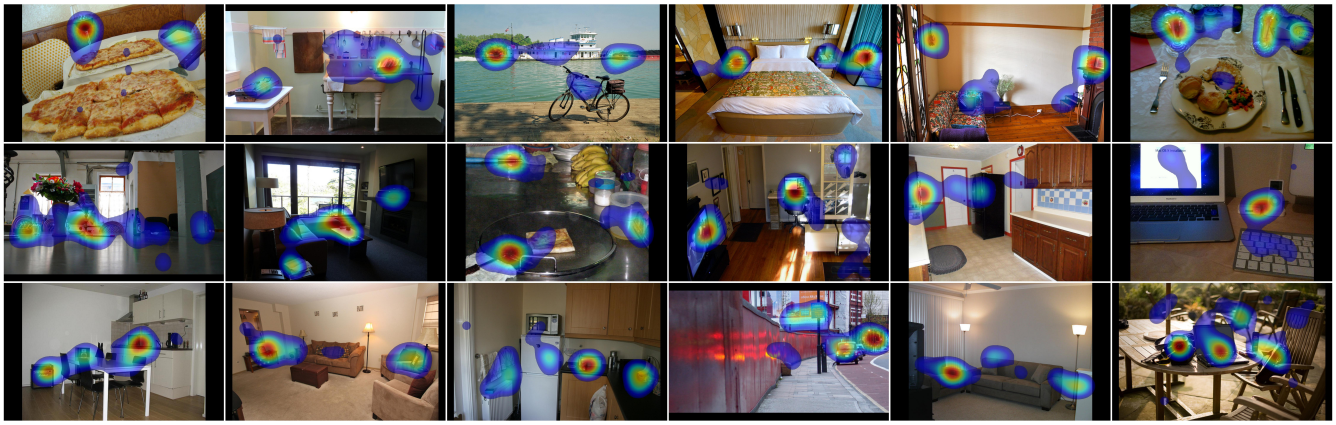

Figure S7. (A). Examples of a target-absent image for each of the 18 target categories. Yellow lines and numbered discs indicate a representative search scanpath from a single participant. From left to right, top to bottom: bottle, bowl, car, chair, (analog) clock, cup, fork, keyboard, knife, laptop, microwave, mouse, oven, potted plant, sink, stop sign, toilet, tv. (B). Examples of fixation density maps for the same target-absent images.

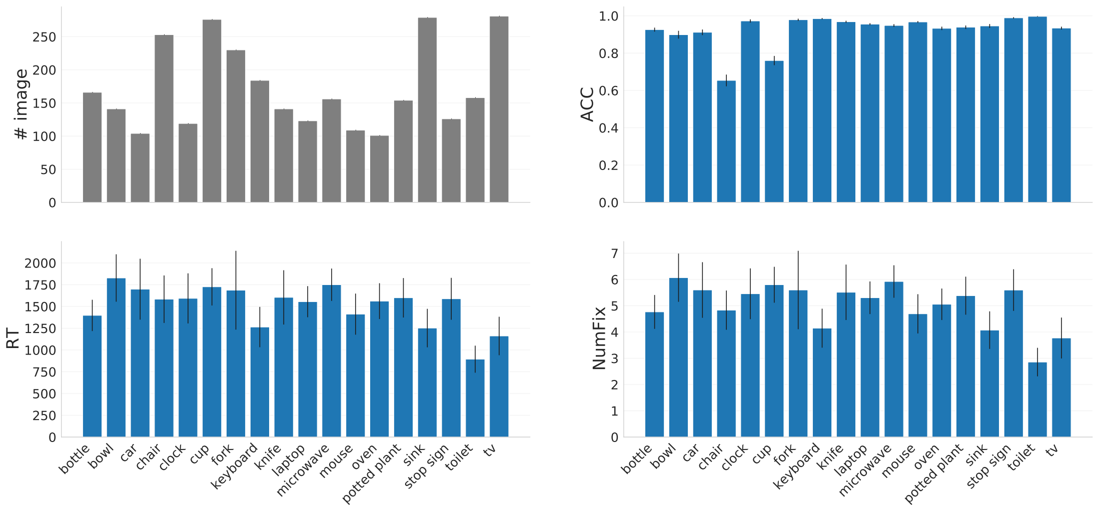

Figure S8. COCO-Search18 analyses for all 18 target categories in target-absent trials. Top: number of images in each category (gray), and response accuracy (ACC). Bottom: reaction time (RT) and number of fixations made before the button press (NumFix). Values are means over 10 participants, and error bars represent standard errors.

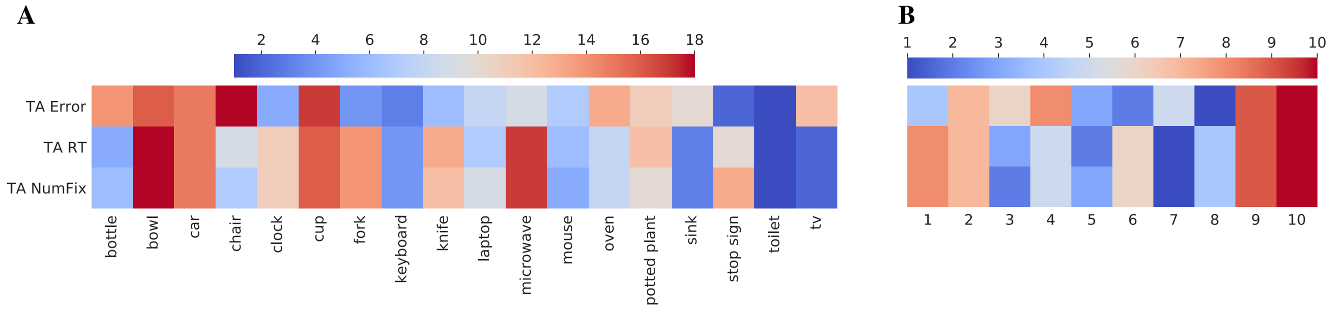

Figure S9. (A). Target-absent data, ranked [1-18] by target category (columns) and averaged over participants, shown for multiple performance measures (rows). These include: response error, reaction time (RT), and number of fixations (NumFix). Redder color indicates higher rank and harder search targets, bluer color indicates lower rank and easier search. (B) Target-absent data, now ranked by participant [1-10] and averaged over target category (columns). Performance measures and color coding are the same as in (A).

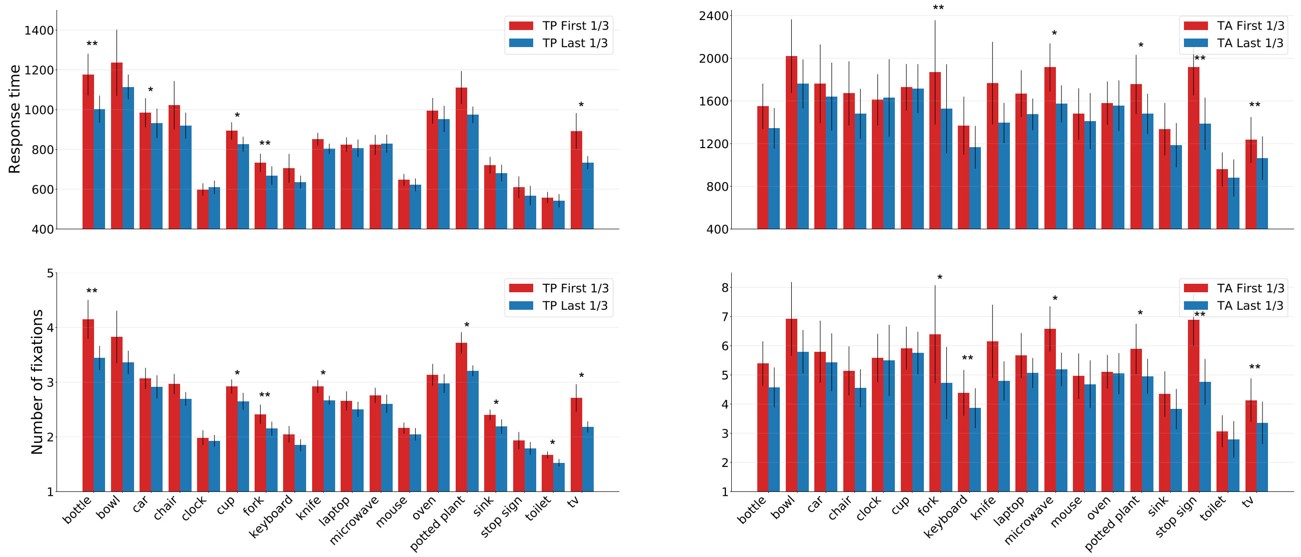

Figure S10. Practice effects, visualized as the difference in search performance between the red (first 1/3 of the trials) and the blue (last 1/3 of the trials) bars, grouped by the 18 target categories. The top row shows response time, and the bottom row shows the number of fixations before the button press. Target-present data are shown on the left, target-absent data are shown on the right. Only correct trials were included. *: $p < .05$, **: $p < .01$

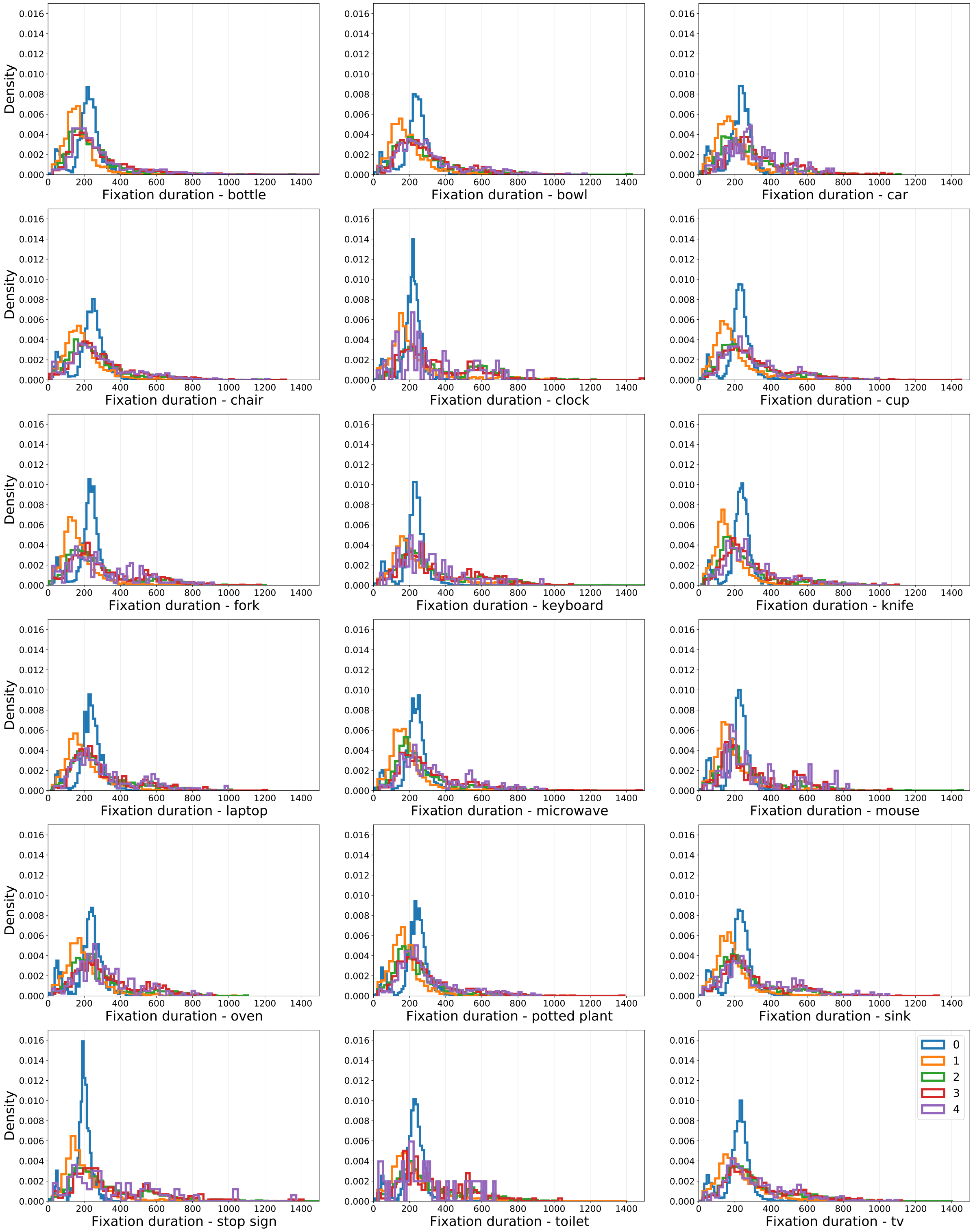

Figure S11. Density distributions of target-present fixation durations, plotted for each of the target categories (bin size = 50ms). The color lines refer to the initial fixation durations (0, blue), followed by the first four new fixations (1-4).

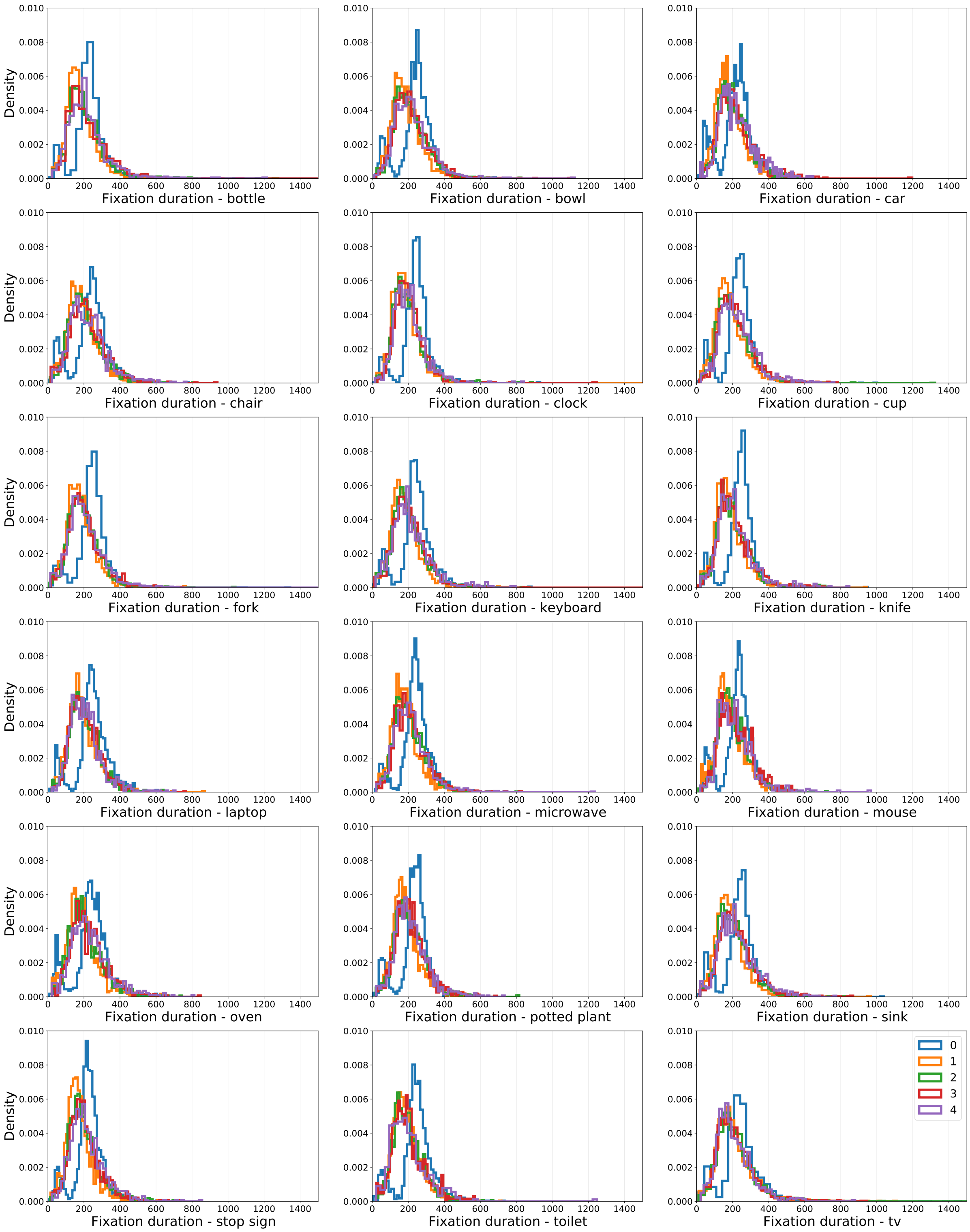

Figure S12. Density distributions of target-absent fixation durations, plotted for each of the target categories (bin size = 50ms). The color lines refer to the initial fixation durations (0, blue), followed by the first four new fixations (1-4).

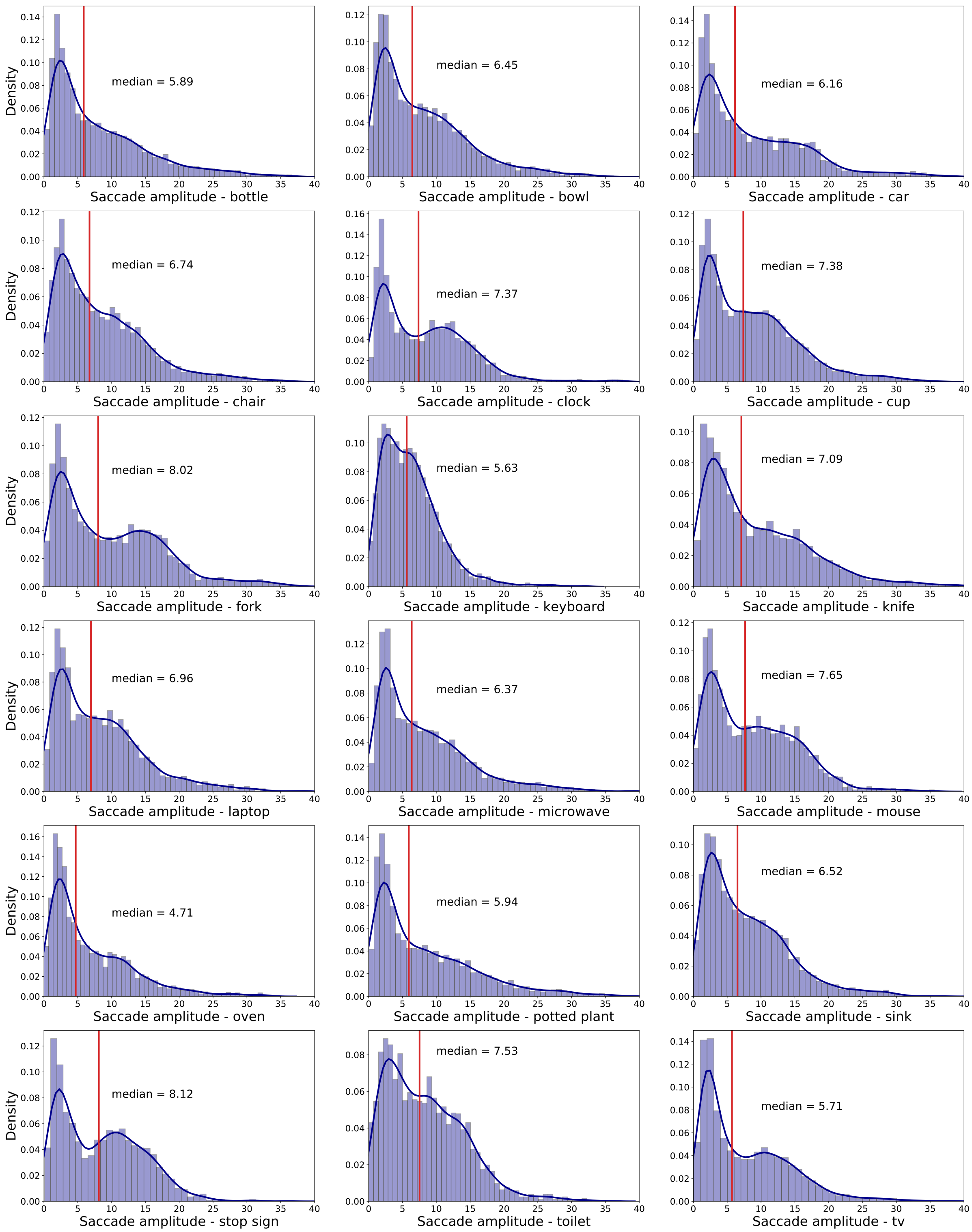

Figure S13. Density distributions of target-present saccade amplitudes (in visual angle), plotted by target category. Red vertical lines indicate median amplitudes. Dark blue lines represent Gaussian kernel density estimates.

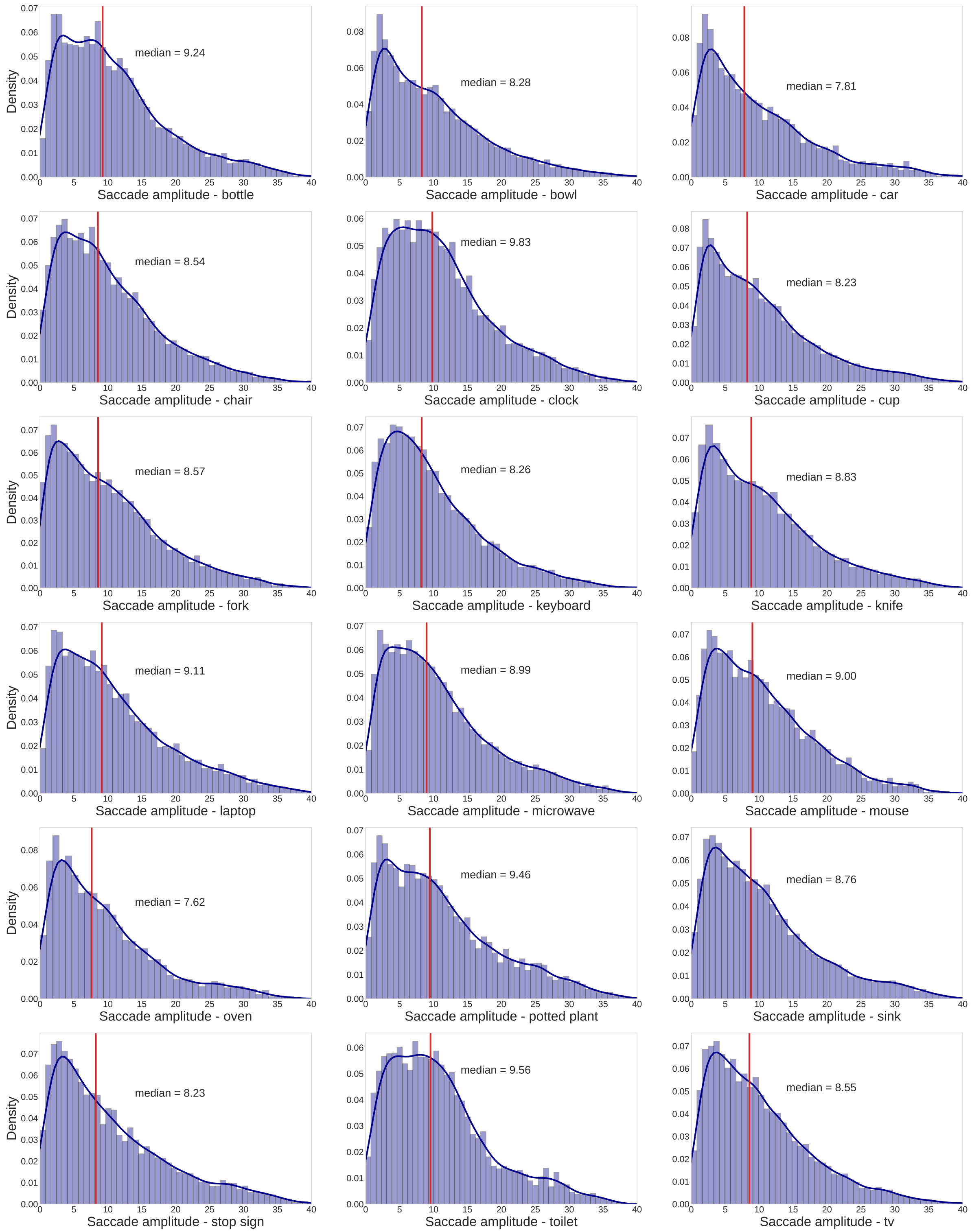

Figure S14. Density distributions of target-absent saccade amplitudes (in visual angle), plotted by target category. Red vertical lines indicate median amplitudes. Dark blue lines represent Gaussian kernel density estimates.

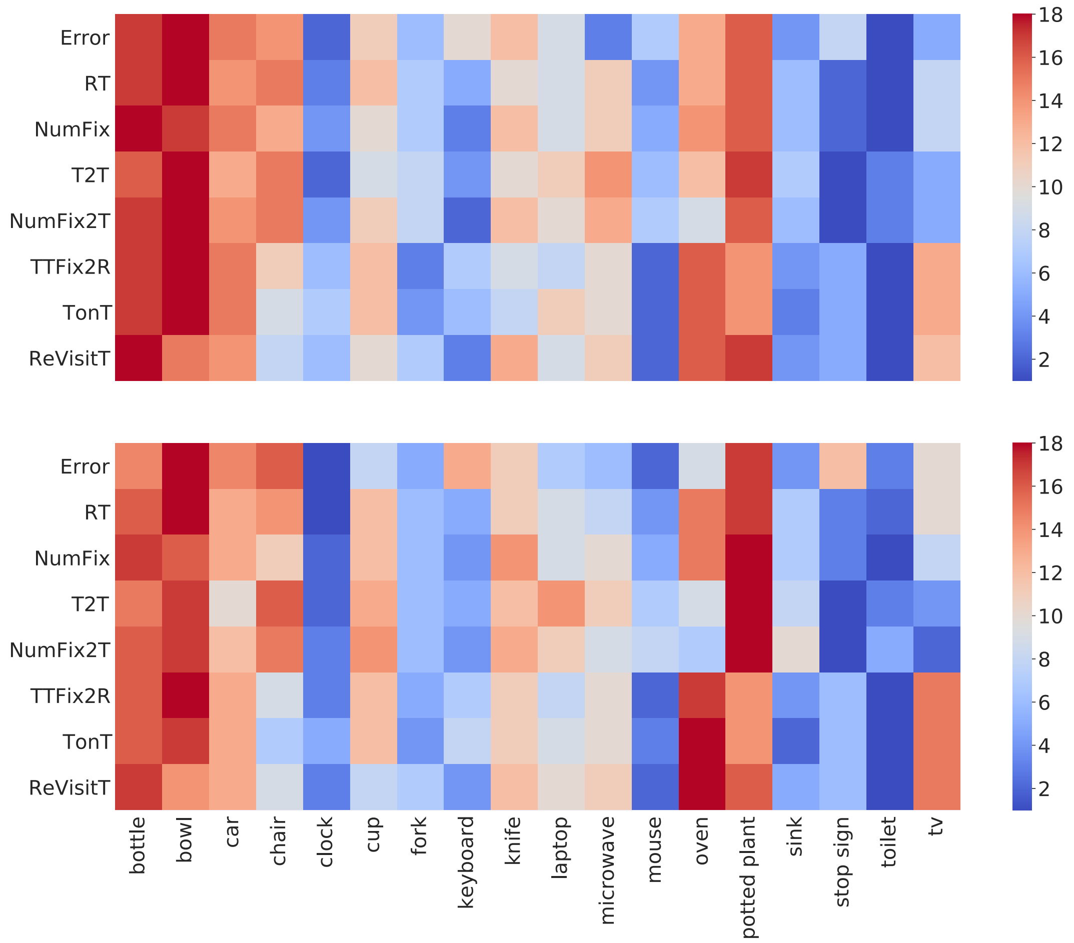

Figure S15. Target-present data, ranked by target category (1-18, columns) and shown for multiple performance measures (rows) in the trainval (top) and test (bottom) COCO-Search18 datasets. Redder color indicates higher rank and harder search targets, bluer color indicates lower rank and easier search. Measuers include: response error, reaction time (RT), number of fixations (NumFix), time to target (T2T), number of fixations to target (NumFix2T), time from first target fixation until response (TTFix2R), time spent fixating the target (TonT), and the number of target re-fixations (ReVisitT).

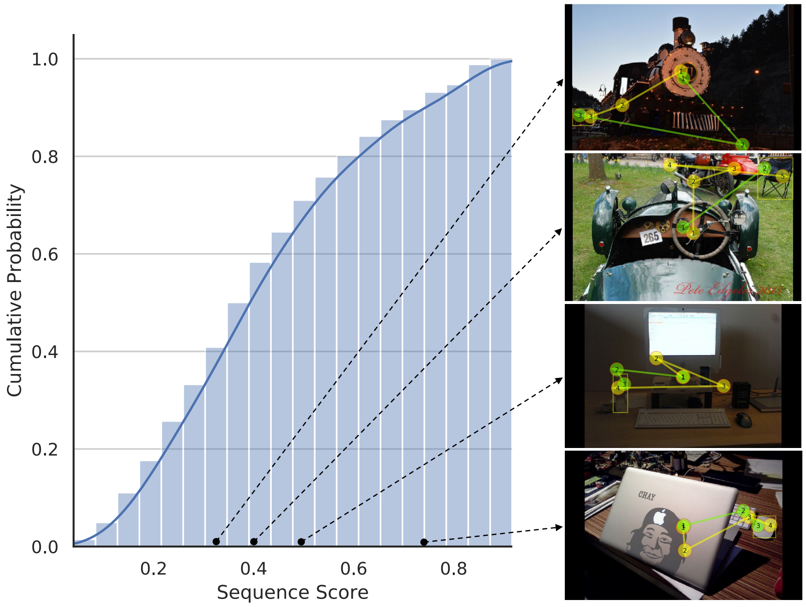

Figure S16. Left: cumulative distribution of average sequence scores computed between each scanpath generated by the IRL model and each behavioral scanpath for the test images of COCO-Search18. Right: Examples illustrating the scanpaths producing four different sequence scores. Behavioral scanpaths are colored in yellow, and the IRL-generated scanpaths are in green. Sequence scores for the four illustrated examples are 0.33, 0.40, 0.50, and 0.75, from top to bottom. Note that these results are from a slightly different version of the IRL model than the one reported here.

A

Participants	Error	RT (ms)	NumFix	T2T (ms)	NumFix2T	TTFix2R (ms)	TonT (ms)	ReVisitT
1	0.06	993.91	2.92	372.81	1.73	769.90	717.79	1.05
2	0.09	878.03	2.81	355.50	1.88	625.61	584.05	0.75
3	0.09	780.30	2.41	349.16	1.76	651.39	622.66	0.58
4	0.10	783.61	2.49	329.89	1.96	489.23	441.90	0.45
5	0.10	761.47	2.63	308.09	1.80	544.66	525.32	0.74
6	0.07	811.03	2.96	352.92	2.12	490.26	460.37	0.71
7	0.08	633.56	2.15	310.47	1.65	429.51	415.33	0.44
8	0.07	713.69	2.44	331.08	1.79	494.28	465.60	0.60
9	0.08	1027.95	2.97	404.65	2.08	564.27	495.76	0.61
10	0.05	825.27	2.37	391.44	1.79	528.57	504.68	0.49
Mean	0.08	820.88	2.61	350.60	1.86	558.77	523.35	0.64

B

Participants	TA Error	TA RT (ms)	TA NumFix
1	0.07	1834.48	5.83
2	0.08	1384.30	4.91
3	0.07	961.80	3.07
4	0.08	1119.09	3.80
5	0.07	954.97	3.21
6	0.06	1336.61	4.92
7	0.07	897.78	2.99
8	0.05	1016.36	3.48
9	0.09	2652.84	8.02
10	0.10	2919.68	9.98
Mean	0.07	1507.79	5.02

Table S1. (A): Detailed behavioral data for 10 participants on 8 measures in target-present (TP) images. (B): Detailed behavioral data for 10 participants on 3 measures in target-absent (TA) images.

	AUC ↑	NSS ↑	CC ↑
Human	0.675	3.396	0.356
Random	0.531	0.280	0.039
Detector-Hi	0.605	1.210	0.163
Detector-Hi-Low	0.575	0.792	0.105
Deep Search-Hi	0.620	1.122	0.153
Deep Search-Hi-Low	0.598	0.864	0.118
IRL-ReT-C	0.595	1.601	0.214
IRL-Hi-Low-C	**0.628**	**1.806**	**0.246**
IRL-Hi-Low	0.621	1.728	0.235

Table S2. Results from models (rows) predicting behavioral fixation-density maps (FDMs) using three spatial comparison metrics (columns), applied to the COCO-Search18 test images. "Human" refers to an oracle method whereby the FDM from half of the searchers was used to predict the FDM from the other half of the searchers. See the supplemental text for additional details about the spatial fixation comparison metrics.

Compared Models	TFP-AUC	Probability Mismatch	Scanpath Ratio	Sequence Score	MultiMatch			
					shape	direction	length	position
IRL-ReT-C vs. IRL-Hi-Low-C	*n.s.*	*n.s.*	*n.s.*	*n.s.*	*n.s.*	*n.s.*	*n.s.*	*n.s.*
IRL-ReT-C vs. IRL-Hi-Low	*n.s.*	*n.s.*	*n.s.*	*n.s.*	*n.s.*	*n.s.*	*n.s.*	*n.s.*
IRL-ReT-C vs. Detector-Hi	*n.s.*	*n.s.*	*n.s.*	*n.s.*	*n.s.*	*n.s.*	*n.s.*	*n.s.*
**IRL-ReT-C** vs. Detector-Hi-Low	*.0017*	*<.001*	*<.001*	*n.s.*	*.005*	*.0686*	*<.001*	*.0039*
**IRL-ReT-C** vs. Deep Search-Hi	*<.001*	*<.001*	*<.001*	*n.s.*	*n.s.*	*<.001*	*n.s.*	*n.s.*
**IRL-ReT-C** vs. Deep Search-Hi-Low	*<.001*	*<.001*	*<.001*	*.0587*	*n.s.*	*<.001*	*n.s.*	*n.s.*
IRL-Hi-Low-C vs. IRL-Hi-Low	*n.s.*	*n.s.*	*n.s.*	*n.s.*	*n.s.*	*n.s.*	*n.s.*	*n.s.*
IRL-Hi-Low-C vs. Detector-Hi	*n.s.*	*n.s.*	*.0653*	*n.s.*	*n.s.*	*n.s.*	*.0235*	*n.s.*
**IRL-Hi-Low-C** vs. Detector-Hi-Low	*<.001*	*<.001*	*<.001*	*n.s.*	*<.001*	*.0515*	*<.001*	*<.001*
**IRL-Hi-Low-C** vs. Deep Search-Hi	*<.001*	*<.001*	*<.001*	*n.s.*	*n.s.*	*<.001*	*n.s.*	*n.s.*
**IRL-Hi-Low-C** vs. Deep Search-Hi-Low	*<.001*	*<.001*	*<.001*	*.0559*	*.0298*	*<.001*	*n.s.*	*.0110*
IRL-Hi-Low vs. Detector-Hi	*n.s.*	*n.s.*	*.0151*	*n.s.*	*n.s.*	*n.s.*	*.0206*	*n.s.*
**IRL-Hi-Low** vs. Detector-Hi-Low	*<.001*	*<.001*	*<.001*	*n.s.*	*<.001*	*.0539*	*<.001*	*<.001*
**IRL-Hi-Low** vs. Deep Search-Hi	*<.001*	*<.001*	*<.001*	*n.s.*	*n.s.*	*<.001*	*n.s.*	*n.s.*
**IRL-Hi-Low** vs. Deep Search-Hi-Low	*<.001*	*<.001*	*<.001*	*.0506*	*n.s.*	*<.001*	*n.s.*	*.0029*
**Detector-Hi** vs. Detector-Hi-Low	*.0019*	*<.001*	*.0086*	*n.s.*	*n.s.*	*n.s.*	*n.s.*	*.0150*
**Detector-Hi** vs. Deep Search-Hi	*<.001*	*<.001*	*<.001*	*n.s.*	*n.s.*	*.0013*	*<.001*	*n.s.*
**Detector-Hi** vs. Deep Search-Hi-Low	*<.001*	*<.001*	*<.001*	*.0755*	*n.s.*	*<.001*	*<.001*	*n.s.*
Detector-Hi-Low vs. Deep Search-Hi	*n.s.*	*n.s.*	*n.s.*	*n.s.*	*<.001*	*n.s.*	*<.001*	*<.001*
Detector-Hi-Low vs. Deep Search-Hi-Low	*n.s.*	*.0275*	*n.s.*	*n.s.*	*.0446*	*n.s.*	*<.001*	*.0511*
Deep Search-Hi vs. Deep Search-Hi-Low	*n.s.*	*n.s.*	*n.s.*	*n.s.*	*n.s.*	*n.s.*	*n.s.*	*.0778*

Table S3. *P* values from post-hoc t-tests (Bonferroni corrected) comparing predictive models (rows), averaged across the 18 target categories, for multiple scanpath metrics (columns). All *dfs* = 34. For decisively significant comparisons, the more predictive model is indicated in boldface.
